# Chemical characteristics of groundwater and surface water affected by human activities in the upper Jinzi River Basin, China

**DOI:** 10.1038/s41598-025-93318-5

**Published:** 2025-03-18

**Authors:** Xiaochen Zhang, Yongcheng Zhang, Yingjia Gui, Ruiyan Sun, Jun Li, Qi Wu, Yongkang Ding, Kang Chen

**Affiliations:** 1 Hebei Province Collaborative Innovation Center for Sustainable Utilization of Water Resources and Optimization of Industrial Structure, Hebei GEO University, Shijiazhuang, 050031 China; 2 Hebei Province Key Laboratory of Sustained Utilization and Development of Water Resources, Hebei GEO University, Shijiazhuang, 050031 China; 3 School of Water Resources and Environment, Hebei GEO University, Shijiazhuang, 050031 China; 4 Hebei Center for Ecological and Environmental Geology Research, Hebei GEO University, Shijiazhuang, 050031 China; 5 Xi’an Center of Mineral Resources Survey, China Geological Survey, Xi’an, 710100 China

**Keywords:** Hydrochemistry, Principal component analysis (PCA), Ion sources, Ion ratio, Chemical weathering

## Abstract

Water resources, as critical ecological and environmental assets, are essential to the social and economic development of countries and regions worldwide. The Jinzi River valley functions as a discharge area for industrial and domestic sewage from mining operations and residential communities along both banks. However, due to human activities, both groundwater and surface water in the region have been contaminated to varying extents. Multiple gold mines are located in the research area. Due to years of unregulated mining, this has had a serious impact on the local ecological environment and water quality. However, research on groundwater and surface water in this region, which are crucial components of the ecological environment, remains limited. This study integrates local socio-economic and hydrogeological conditions, employing methods such as multivariate statistical analysis, the Piper trilinear diagram, the Gibbs model, and ionic ratio relationships to analyze the characteristics and origins of major ions in the region. (1) The primary hydrochemical type of surface water was HCO_3_-Ca·Na, while groundwater was predominantly of the HCO_3_-Ca·Mg type. This hydrochemical pattern was consistent across the region, with ion concentrations significantly higher in areas dominated by carbonate rock compared to those with silicate rock. (2) Using principal component analysis, water–rock interaction modeling, and ion source analysis, it was determined that groundwater chemistry was primarily influenced by the weathering of diorite and carbonate rock, along with inputs from domestic and agricultural wastewater. In contrast, surface water chemistry was largely controlled by the weathering of carbonate rocks and the discharge of industrial wastewater. (3) Components such as SO_4_^2−^, NO_3_^−^, Cl^−^, and total dissolved solids in surface water exhibited a pronounced sensitivity to human activities, with their concentrations significantly exceeding those in groundwater. This indicates that surface water is more heavily impacted by human activities, particularly from industrial, agricultural, and domestic wastewater sources.

**Supplementary Information:**

The online version contains supplementary material available at 10.1038/s41598-025-93318-5.

## Introduction

Water resources are vital for industrial and agricultural production and serve as a fundamental ecological and environmental factor, playing an essential role in the socio-economic development of countries and regions worldwide^1^. In the Jinzi River Valley, industrial and domestic wastewater from nearby mining operations and local communities is discharged into the river. Consequently, human activities have led to varying degrees of contamination in both groundwater and surface water^2^.

The Daping gold deposit, situated in Yuanyang County, Yunnan Province, China, is located within the Jinzi River Basin. This polymetallic sulfide quartz vein ore body is abundant in gold, with associated minerals including lead, silver, and copper. Mining activities in the area have a history of over 300 years, dating back to the late Ming Dynasty^3^. However, the predominantly small-scale and unregulated nature of mining in the region has caused significant degradation of the local ecological environment. Although shallow gold deposits have been depleted, deep mining operations persist. In addition to mining, water remains vital for the daily needs of local residents. Despite the importance of groundwater and surface water to the local ecosystem, research on their current state is limited. Comprehensive studies on the chemical characteristics and underlaying causes of surface and groundwater contamination under the influence of human activities are essential for ensuring the sustainable management and utilization of water resources in the region.

In natural waters, major elements are widely distributed and abundant, forming the primary ionic components of the water. The ionic composition is a valuable indicator of the rock lithology and climatic conditions of the regions through which the water flows. It is extensively used to identify key processes controlling the chemical composition of water, including rock weathering, evaporation and crystallization, land use changes, precipitation, and human activities^4–7^. Understanding the chemical characteristics of rivers is essential for accurately identifying the sources of ions and the processes influencing water chemistry^8^. Since the twentieth century, researchers have extensively studied the water chemistry of major rivers worldwide, aiming to understand the primary mechanisms that control water chemistry in different river basins^9–12^.

Currently, multivariate statistical analysis methods are widely employed as primary tools for studying the chemical characteristics of water. Many studies combine local geological conditions, climatic factors, and land use patterns to examine the hydrochemical evolution and processes within drainage basins. Techniques such as cluster analysis (CA) and principal component analysis/factor analysis (PCA) are commonly utilized for these investigations^13–18^. The relationship between ionic ratios is commonly used to understand the formation and circulation of groundwater and surface water. Researchers have applied ion ratio relationships alongside methods such as Piper trilinear diagrams, Gibbs models, and multivariate statistical analysis to analyze hydrochemical data from both groundwater and surface water. These approaches help identify the sources of major ions and the factors influencing water quality^19–23^.

This study aims to systematically analyze the chemical characteristics and spatial distribution of major ions in groundwater and surface water within the Jinzi River Basin. By employing multivariate statistical analysis and ion ratio methods, the research explores the underlying causes of water chemistry variations. Through an analysis of the key ionic characteristics in both surface and groundwater and the factors influencing them, this study provides essential data to support water pollution prevention and control, as well as the sustainable development, utilization, and protection of water resources in the region.

## Materials and methods

### Study area

**Fig. 1:**
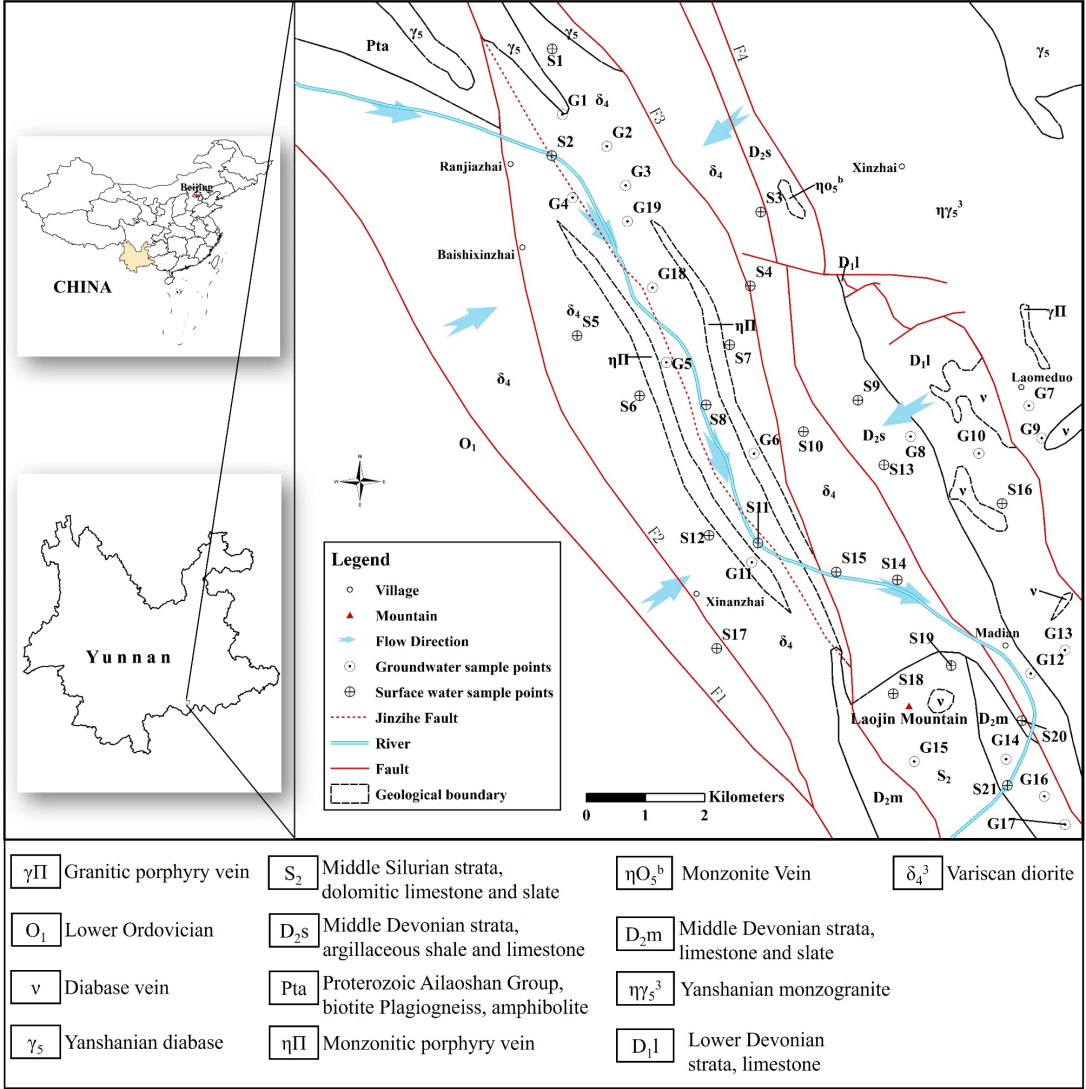
Distribution of water samples in the Jinzi River Basin (Modified from Ge et al.^24^).

The study area is located in Daping Township, Yunnan Province, China, with geographical coordinates ranging from 103° 02′ 00″ to 103° 09′ 00″ east longitude and 22° 50′ 00″ to 22° 58′ 00″ north latitude. The area spans approximately 174 km^2^ (Fig. 1).

The climate in the area is classified as a southern tropical monsoon climate. Based on meteorological data from the past 30 years, the region experiences an average annual temperature of 18.1 °C, average annual precipitation of 2331.3 mm, and average annual evaporation of 1388.9 mm. The rainy season typically lasts from May to November, with the heaviest precipitation occurring between May and August.

The area is characterized by middle to low mountain landforms, shaped by tectonic erosion, with mountains predominantly extending in a northwest-southeast direction. Groundwater in the region is primarily found in Quaternary Holocene alluvial-fluvial gravel and pebble layers, semi-weathered bedrock fissure layers composed of igneous and metamorphic rocks, as well as in the carbonate rock dissolution fissure zones of the Jinzi River. Atmospheric precipitation is the primary source of groundwater recharge in the area. Groundwater from the diorite flows into the Jinzi River from both the east and west sides, typically exiting the Jinzi River Valley in a northwest (NW) to southeast (SE) direction. The groundwater in the Laojinshan carbonate rock is deeply buried and only emerges as spring water in the lower parts of the surrounding area, discharging radially in all directions.

The region is characterized by well-developed fault structures, with early faults predominantly trending NW. The primary faults in the area include the Sanjiahe Fault (F1), Ranjiazhai-Xinanzhai Fault (F2), Xiaoxinjie Fault (F3), and Xiaozhai-Jinping Fault (F4). F1 and F4 act as boundary faults, controlling the distribution of Variscan diorite in the region. The Jinzi River roughly follows the erosion and incision along F3, leading to the formation of the region’s lowest gorge.

### Sampling and measurements

Before collecting the samples, the sample bottles were immersed in dilute nitric acid and thoroughly cleaned with deionized water. The bottles were then pre-rinsed three times with the water sample to be collected. Ensure that the sample bottle is completely filled with the water sample during collection. Two bottles of water were collected from each sampling point, sealed, and stored in polyethylene sample bottles at 4 °C, away from light. The pH value was measured on-site. One of the water sample was acidified with nitric acid to achieve a pH ≤ 2, for testing metal cations such as K^+^, Na^+^, Ca^2+^, and Mg^2+^. The other bottle, which contains the original sample, was used for testing chemical parameters such as HCO_3_^−^, Cl^−^, SO_4_^2−^, F^−^, NO_3_^−^, and TDS. The analysis was conducted by the Kunming Mineral Resources Testing Center, which operates under the Ministry of Natural Resources. The water samples were analyzed in accordance with the relevant standards for drinking water quality (GB5750) and groundwater quality (GB/T14848). During sampling, the locations of the corresponding sampling points were marked on a topographic map. Surface water samples were labeled S1 to S21, and groundwater samples were labeled G1 to G19. The locations of the sampling points are shown in Fig. 1. A total of 40 sets of water samples were collected across the region, including 19 sets of groundwater samples and 21 sets of surface water samples.

### Data analysis methods

#### Cluster analysis

Cluster analysis is a multivariate statistical technique commonly employed in classification studies. Based on the similarity between samples or indicators, those with a higher degree of similarity are grouped into one category. The remaining samples or indicators, which exhibit greater similarity to each other, are placed into a separate category. This process is repeated iteratively until all samples or indicators have been categorized^25^. Cluster analysis is generally categorized into Q-type and R-type, depending on the research object. Q-type cluster analysis is utilized to classify samples, while R-type cluster analysis is employed to classify indicators. Assuming other factors are negligible, groundwater located in closer proximity is likely to share the same recharge source or exhibit stronger hydraulic connections. In contrast, greater geographic distance typically corresponds to more distinct chemical compositions and weaker hydraulic connections^26^.

In this study, the Ward’s method algorithm, based on the sum of squares, and Euclidean distance were used to conduct Q-type cluster analysis on 11 water quality indicators, including Na^+^, K^+^, Ca^2+^, Mg^2+^, Cl^−^, SO_4_^2−^, HCO_3_^−^, NO_3_^−^, F^−^, TDS, and pH, in water samples collected from the study area. The results of the cluster analysis were visualized as a cluster dendrogram.

#### Principal component analysis

PCA is a statistical technique used to retain essential information from the original dataset while reducing the number of analysis indicators. It eliminates correlations among various indicators and transforms multiple indicators into a smaller set of comprehensive indicators, referred to as principal components. Each principal component captures a significant portion of the information contained in the original indicators^27,28^.

The principle of PCA can be described as follows: Suppose a dataset consists of *n* samples, each characterized by *m* indicators *X*_1_, *X*_2_, …, *X*_*m*_. The matrix representation of these data is given by:

1 $$X = \left( {\begin{array}{*{20}c} {x_{11} } & {x_{12} } & \cdots & {x_{1m} } \\ {x_{21} } & {x_{22} } & \cdots & {x_{2m} } \\ \vdots & \vdots & {x_{ij} } & \vdots \\ {x_{n1} } & {x_{n2} } & \cdots & {x_{nm} } \\ \end{array} } \right) = \left( {\begin{array}{*{20}c} {X_{1} ,} & {X_{2} ,} & { \cdots ,} & {X_{m} } \\ \end{array} } \right)$$

where *X*_*k*_ = (*X*_*1k*_, *X*_*2k*_, …, *X*_*nk*_) for *k* = 1,2,3, …, *m*, *n* represents the number of water samples collected, indexed by *i* = 1, 2… n, *m* represents the total number of characteristic indicators, indexed by *j* = 1, 2… m, and *x*_*ij*_ denotes the *j*th indicator value for the *i*th sample.

Next, let *F*_*j*_ (j = 1, 2, …, m) be the composite of the *m* indicators, expressed as:

2 $$\left\{ {\begin{array}{*{20}c} {F_{1} = a_{11} X_{1} + a_{12} X_{2} + \cdots + a_{1m} X_{m} } \\ {F_{2} = a_{21} X_{1} + a_{22} X_{2} + \cdots + a_{2m} X_{m} } \\ \vdots \\ {F_{m} = a_{m1} X_{1} + a_{m2} X_{2} + \cdots + a_{mm} X_{m} } \\ \end{array} } \right.$$

where *F*_m_ is the *m*th principal component, and *a* represents the principal component coefficients.

Additionally, PCA must satisfy the following conditions:

- a_*j*1_^2^ + a_*j*2_^2+^… + a_*jm*_^2^ = 1 for *j* = 1, 2, …, *m*;
- *F*_*j*_ (*j* = 1, 2, …, *m*) are uncorrelated with each other;
- The variance of *F*_*j*_ (*j* = 1, 2, …, *m*) decreases in order of importance.

In this study, factor analysis of Na^+^, K^+^, Ca^2+^, Mg^2+^, Cl^−^, SO_4_^2−^, HCO_3_^−^, NO_3_^−^, F^−^, TDS, and pH in groundwater and surface water samples was performed using generalized principal component extraction and maximum variance orthogonal rotated factor analysis methods. To mitigate the impact of missing data, any missing values were substituted with the mean values of their respective indicators.

## Results and discussion

### Water chemical characteristics

#### Characteristics of water chemical composition

The chemical parameters of each water sample are presented in Table 1. The groundwater is generally weakly alkaline, with pH values ranging from 7.11 to 7.84, and an average of 7.43. It is characterized as low-salinity fresh water, with salinity levels ranging from 149.44 to 380.88 mg/L. The coefficient of variation (CV) is 21.58%, indicating relatively stable concentrations compared to surface water. Except for Mg^2+^ and F^−^, the standard deviation of each component’s concentration is smaller than that of surface water, suggesting greater stability. The concentration order of the main cations is Ca^2+^ > Na^+^ > Mg^2+^ > K^+^, and for the main anions, the order is HCO_3_^−^ > SO_4_^2−^ > Cl^−^ > NO_3_^−^ > F^−^. The primary cations are Ca^2+^ and Mg^2+^, with a percentage variation range of 41.85–61.54% and 9.65–41.20% in milligram equivalent concentration, respectively. HCO_3_^−^ is the predominant anion, with a variation range of 35.78–90.93%.

**Table 1:** Statistics of major ion mass concentration/(mg/L).

Types	Items	K^+^	Na^+^	Ca^2+^	Mg^2+^	HCO_3_^−^	Cl^−^	SO_4_^2−^	F^−^	NO_3_^−^	TDS	pH
Ground-water	Mean	3.79	17.49	36.58	10.23	131.34	6.45	33.14	0.08	2.73	239.01	7.43
	Max	11.86	36.12	56.31	23.22	169.14	24.11	148	0.2	8.55	380.88	7.84
	Min	0.85	4.23	19.31	4.51	97.7	1.13	6.78	0.07	0.38	149.44	7.11
	Std	2.44	9.29	12.53	4.6	19.91	6.16	32.28	0.03	1.85	51.59	0.22
	CV/%	64.44	53.12	34.26	45	15.16	95.5	97.41	37.16	67.84	21.58	3.01
Surface water	Mean	4.62	24.04	42.77	9.57	142.52	7.45	62.93	0.07	5.11	293.9	7.45
	Max	12.36	120.35	136.27	19.55	221.4	32.46	296.41	0.12	23.41	602.8	8.15
	Min	1.23	4.1	21.34	4.15	60.84	0	5.11	0.02	1.04	125.37	7.16
	Std	3.07	22.49	23.91	4.45	38.16	7.31	90.94	0.02	6.29	140.22	0.22
	CV/%	66.36	93.56	55.9	46.52	26.78	98.08	144.5	34.3	123.03	47.71	2.99

Std: Standard deviation; CV: Coefficient of variation.

Surface water is also slightly alkaline, with pH values ranging from 7.16 to 8.15, and an average of 7.45. TDS values range from 125.37 to 602.8 mg/L, with a CV of 47.71%. The concentrations of each component in surface water are slightly higher than those in groundwater. The concentration order of conventional anions and cations in surface water mirrors that of groundwater. The primary cation is Ca^2+^, accounting for approximately 50% of the total cations, while HCO_3_^−^ is the predominant anion, comprising 35–90% of the total anions. The maximum concentrations of SO_4_^2−^ and NO_3_^−^ in surface water are 296.41 mg/L and 23.41 mg/L, respectively, indicating localized pollution by SO_4_^2−^ and NO_3_^−^. Specifically, the Jinzi River, situated lower than the surrounding villages and mines, acts as a discharge area for industrial and domestic wastewater, contributing to this pollution.

The CV reflects the degree of spatial dispersion of hydrochemical parameters^29,30^. As shown in Table 1, with the exception of Mg^2+^, TDS, and pH in groundwater, as well as pH and HCO_3_^−^ in surface water, the remaining values exhibit significant variations. Notably, the CVs of SO_4_^2−^ and NO_3_^−^ in surface water exceed 1, while Na^+^ and Cl^−^ approach 1. This suggests that surface water in certain areas has been contaminated to varying degrees with nitrate, sulfate, and chloride. The severity of pollution is reflected in the increasing disparity in nitrate and sulfate ion concentrations between wastewater and clean water. The CVs of Cl^−^ and SO_4_^2−^ in groundwater are also close to 1, indicating similar contamination levels in groundwater. Additionally, the CV values for other ions in water are generally around 0.5 or lower.

#### Types of water chemistry

The hydrochemical types of groundwater and surface water are illustrated in Supplementary Fig. S1. In this figure, most of the water sample points are located in areas where weak acid ions predominate over strong acid ions. Additionally, a smaller number of sample points are found in regions where strong acid ions exceed weak acid ions. The predominant hydrochemical types are HCO_3_-Ca·Mg and HCO_3_·SO_4_-Ca·Na, which account for 42.11% and 26.32% of the groundwater samples, respectively. Additionally, a smaller number of samples exhibit the following types: SO_4_·HCO_3_-Ca·Na, HCO_3_·SO_4_-Ca·Na·Mg, and HCO_3_·SO_4_-Ca. The hydrochemical types are highly complex. The dominant hydrochemical type in surface water is HCO_3_-Ca·Na, which accounts for 57.17% of the samples, followed by SO_4_·HCO_3_-Ca and HCO_3_-Ca·Mg, each representing 14.29% of the total samples. The ions HCO_3_^−^, Na^+^, Ca^2+^, and Mg^2+^ are primarily derived from the dissolution of carbonate and silicate rocks, indicating that the area is predominantly influenced by the weathering and dissolution of these rocks.

**Fig. 2:**
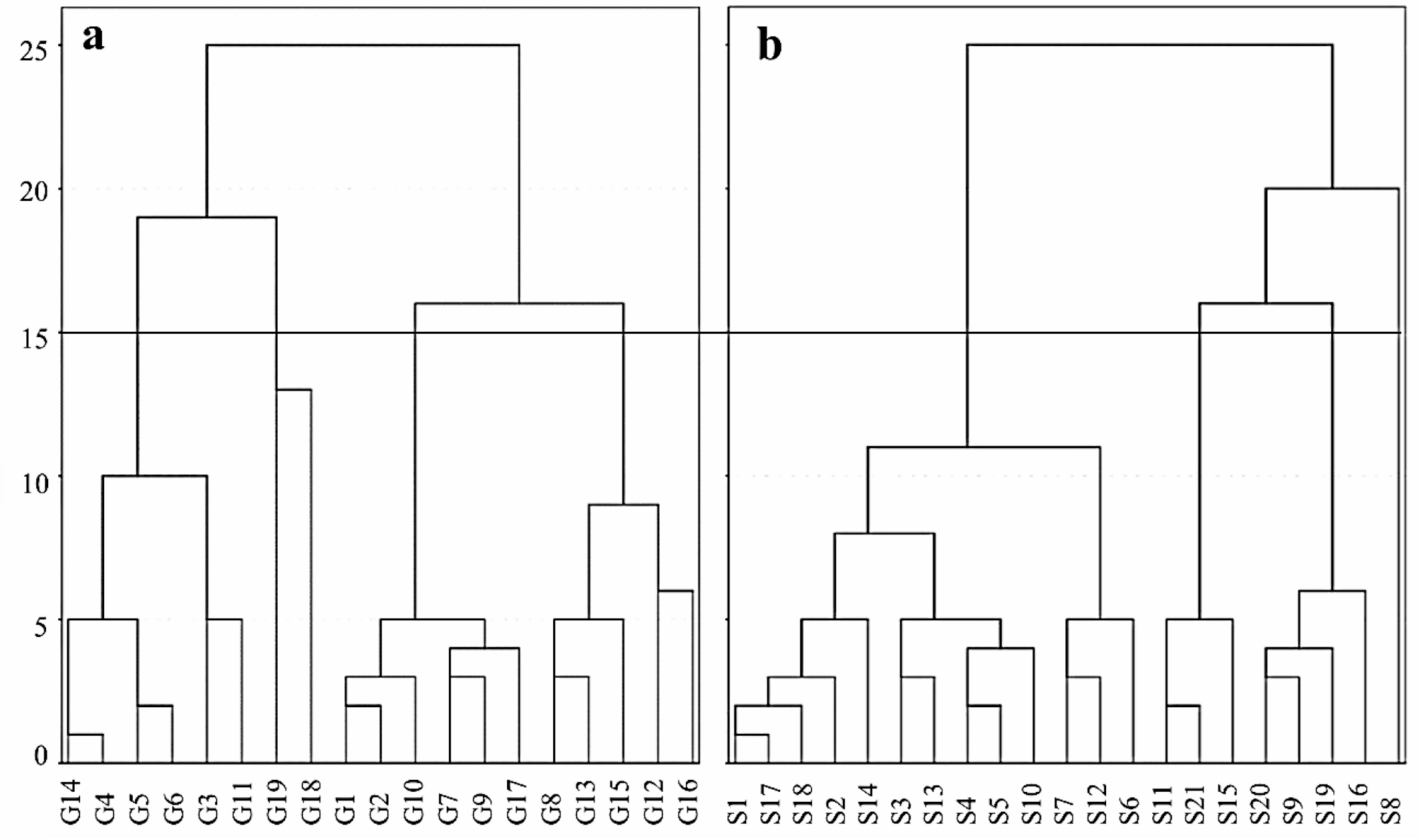
Cluster analysis of water chemical indices for different types: (**a**) groundwater; (**b**) surface water in the area.

Cluster analysis, which involves grouping data with strong similarities into the same class or cluster, has been widely applied in water chemistry research^31–35^. The 11 parameters listed in Table 1 were selected for analysis. The Ward method was used to conduct systematic cluster analysis on groundwater and surface water samples from the study area, with an inter-class distance of 15. The results of the cluster analysis are presented in Fig. 2. The 19 groups of groundwater samples were classified into four categories: HCO_3_·SO_4_-Ca·Na, SO_4_·HCO_3_-Ca·Na, HCO_3_-Ca·Mg(I), and HCO_3_-Ca·Mg(II). Similarly, the 21 groups of surface water samples were clustered into four categories: HCO_3_-Ca·Na, HCO_3_-Ca·Mg, SO_4_·HCO_3_-Ca, and SO_4_·HCO_3_-Ca·Na. These results align with the analysis from the Piper diagram.

**Fig. 3:**
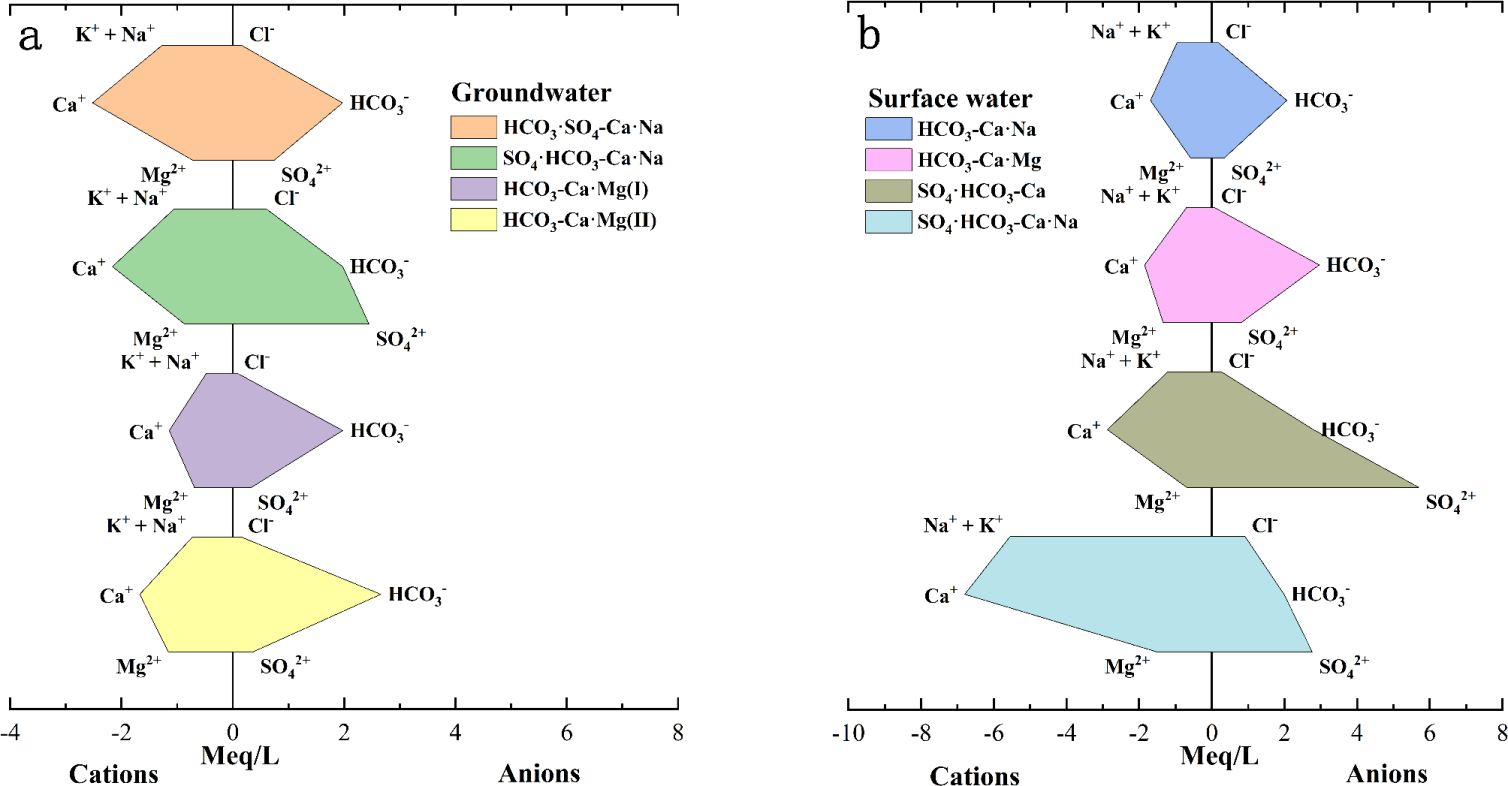
Stiff diagrams of chemical components: (**a**) groundwater; (**b**) surface water.

According to Fig. 2 and Supplementary Table S1, significant differentiation is observed among the physicochemical indices of the four types of groundwater and four types of surface water. This differentiation is clearly illustrated in the Stiff diagram (Fig. 3). The concentrations of various components in groundwater, such as HCO_3_·SO_4_-Ca·Na, HCO_3_-Ca·Mg(I), and HCO_3_-Ca·Mg(II), are relatively stable (Table S1).

Approximately 32% of the groundwater samples are classified as HCO_3_·SO_4_-Ca·Na, with an average pH of 7.56 and an average TDS of 252.77 mg/L. The average concentrations of K^+^, Na^+^, and Ca^2+^ are the highest among the clusters. The dominant cations in this group are Ca^2+^ and Na^+^, accounting for 48.38–61.03% and 23.89–31.03% of the total cations, respectively. The dominant anions in this group are HCO_3_^−^ and SO_4_^2−^, accounting for 69.53–77.1% and 20.98–22.37% of the total anions, respectively. This type is primarily distributed along both sides of the Jinzi River, where diorite is widely present. Therefore, it is speculated that the HCO_3_·SO_4_-Ca·Na type may be associated to the dissolution of diorite (Table S1, Fig. S2).

The smallest cluster, SO_4_·HCO_3_-Ca·Na, has an average pH of 7.49 and an average TDS of 338.67 mg/L. The average concentrations of Cl^−^, SO_4_^2−^, F^−^, and NO_3_^−^ are the highest among the clusters. The dominant cations in this group are Ca^2+^ and Na^+^, accounting for 54.71–55.18% and 22.37–37.48% of the total cations, respectively. The dominant anions are SO_4_^2−^ and HCO_3_^−^, accounting for 38.41–48.70% and 42.58–49.18% of the total anions, respectively. This type is distributed between the Jinzi River and a granite porphyry vein, an area where industrial mining activities are taking place. It is speculated that the SO_4_·HCO_3_-Ca·Na type may be influenced by these industrial activities (Fig. S2).

Approximately 32% of the samples are classified as HCO_3_-Ca·Mg(I), with an average pH of 7.33 and an average TDS of 182.15 mg/L. The dominant cations in this group are Ca^2+^ and Mg^2+^, accounting for 44.41–63.56% and 16.28–27.7% of the total cations, respectively. The dominant anion is HCO_3_^−^, which accounts for 75.18% to 93.19% of the total anions. This type is mainly distributed in the diorite and granite areas in the northwest (Table S1, Fig. S2).

Approximately 26% of the samples are classified as HCO_3_-Ca·Mg(II), with an average pH of 7.37 and an average TDS of 250.87 mg/L. The average concentrations of HCO_3_^−^ and Mg^2+^ are the highest among the samples. The dominant cations are Ca^2+^ and Mg^2+^, accounting for 44.23–61.73% and 11.66–27.31% of the total cations, respectively. The dominant anion is HCO_3_^−^, which makes up 83.63–90.22% of the total anions. This type is primarily found in the carbonate rock zone downstream and may be influenced by the dissolution of carbonate rocks (Table S1, Fig. S2).

The milligram equivalent concentrations of each component in HCO_3_-Ca·Mg(I) and HCO_3_-Ca·Mg(II) are presented in Table 2 and Fig. 3. Although both are HCO_3_-Ca·Mg type, their hydrochemical compositions differ significantly in terms of concentration. HCO_3_-Ca·Mg(I) is primarily found in diorite and granite areas, with relatively lower concentrations of each component. In contrast, HCO_3_-Ca·Mg(II) is mainly distributed in carbonate rock areas, exhibiting higher concentrations of each component, indicating a greater influence from carbonate rock weathering compared to silicate rock weathering.

**Table 2:** Statistics analysis of physical and chemical characteristics of HCO_3_-Ca·Mg(I) and HCO_3_-Ca·Mg(II) types (average)/(meq/L).

	Indicators	Na^+^ + K^+^	Ca^2+^	Mg^2+^	HCO_3_^**−**^	Cl^−^	SO_4_^2−^	NO_3_^−^
HCO_3_-Ca·Mg(I)	Max	0.75	1.27	0.88	2.11	0.12	0.74	0.06
	Min	0.28	0.97	0.54	1.60	0.04	0.14	0.01
	Mean	0.48	1.15	0.70	1.97	0.08	0.32	0.03
HCO_3_-Ca·Mg(II)	Max	1.28	2.46	1.94	2.77	0.29	0.42	0.08
	Min	0.37	1.37	0.70	2.57	0.06	0.27	0.01
	Mean	0.73	1.68	1.18	2.66	0.15	0.37	0.05

More than half of the surface water samples are classified as HCO_3_-Ca·Na, with an average pH of 7.36 and a mean TDS of 214.17 mg/L. Among the samples, NO_3_^−^ exhibits the highest average concentration. The predominant cations are Ca^2+^ and Na^+^, contributing 44.11–63.66% and 20.98–33.09% of the total cations, respectively. HCO_3_^−^ is the dominant anion, accounting for 64.52% to 83.42% of the total anions. This water type is widely distributed within the diorite formations of the area. The terrain is low and flat, situated in a groundwater discharge zone. The mean values closely align with those of groundwater, indicating a potential influence from groundwater (Table S1, Fig. S2).

Approximately 19% of the samples are classified as HCO_3_-Ca·Mg, with an average pH of 7.11 and a mean TDS of 291.84 mg/L. HCO_3_^−^ and F^−^ exhibit the highest average concentrations. The predominant cations are Ca^2+^ and Mg^2+^, contributing 45.31–60.42% and 14.89–29.15% of the total cations, respectively. HCO_3_^−^ is the dominant anion, comprising 74.32% to 87.44% of the total anions. This water is located in the southeastern carbonate zone of the area and may be influenced by carbonate dissolution (Table S1, Fig. S2).

Approximately 14% of the samples are classified as SO_4_·HCO_3_-Ca, with a mean pH of 7.45 and a mean TDS of 548.83 mg/L. SO_4_^2−^ exhibits the highest average concentration, while the dominant cation is Ca^2+^, accounting for 58.5% to 62.87% of the total cations. The dominant anions are SO_4_^2−^ and HCO_3_^−^, contributing 54.28–61.9% and 31.77–39.13% of the total anions, respectively. This water type is distributed downstream in the area and partially reflects the influence of human activities (Fig. S2).

The SO_4_·HCO_3_-Ca·Na water type is characterized by a mean pH of 7.52 and a mean TDS of 573.83 mg/L. The predominant cations are Ca^2+^ and Na^+^, contributing 47.42% and 41.88% of the total cations, respectively. The dominant anions are SO_4_^2−^ and HCO_3_^−^, comprising 45.93% and 42.05% of the total anions, respectively. This water type is distributed in the central part of the area, where the average concentrations of K^+^, Na^+^, Ca^2+^, Mg^2+^, and Cl^−^ are the highest (Table S1, Fig. S2).

### Analysis of the main causes of the evolution of water chemistry

#### Chemical correlation analysis

The correlation analysis of hydrochemical components provides valuable insights into the sources and interrelationships of ions in water^36,37^. Pearson correlation coefficients were calculated for the chemical parameters of all water samples, with the results presented in Supplementary Table S2 (available online).

A significant positive correlation is observed between TDS and Na^+^, Ca^2+^, Cl^−^, SO_4_^2−^, and F^−^ in groundwater, with correlation coefficients of 0.705, 0.673, 0.748, 0.811, and 0.654, respectively. This indicates that these ions collectively contribute to the TDS in the water. Additionally, there is a strong positive correlation between Na^+^ and Ca^2+^ (0.785), suggesting a potential link between these ions and water hardness, which is usually dominated by calcium and magnesium ions. Both ions may be influenced by similar sources and environmental factors. A moderate positive correlation is observed between Na^+^ and SO_4_^2−^ (0.492), suggesting that they may originate from similar sources. The dissolution of sulfate salts in water could contribute to an increase in sodium. The strong positive correlation between Cl^−^ and SO_4_^2−^ (0.869) likely indicates a common origin, particularly the discharge of industrial wastewater. Cl^−^ and NO_3_^−^ exhibit a significant correlation (0.47), likely reflecting the impact of agricultural pollution on groundwater. F^−^ shows a strong correlation with both Cl^−^ and SO_4_^2−^, suggesting that its presence may be linked to anthropogenic pollution. Additionally, the positive correlation between NO_3_^−^ and pH (0.461) indicates that higher nitrate concentrations are associated with increased groundwater acidity, which may be attributed to the use of agricultural fertilizers or the discharge of wastewater.

The correlation matrix of chemical components in surface water reveals significant positive correlations between TDS and Na^+^, Ca^2+^, HCO_3_^−^, Cl^−^, SO_4_^2−^, and NO_3_^−^, with correlation coefficients of 0.567, 0.752, 0.549, 0.583, 0.908, and 0.757, respectively. Na^+^ and Cl^−^ exhibit a strong correlation (0.839), while the correlation coefficients of Na^+^ with other major anions are notably smaller. The correlation coefficient between K^+^ and Cl^−^ is 0.641, indicating a significant correlation. Considering that Na^+^ and K^+^ usually have the same source, this suggests the possible dissolution of salt rock. The correlation between Ca^2+^ and SO_4_^2−^ is relatively significant (0.511), likely linked to the dissolution of substances such as gypsum. The strong correlation between NO_3_^−^ and SO_4_^2−^ (0.932) suggests a common origin, most likely related to human activities. The correlation between Na^+^ and Ca^2+^ is 0.911, indicating a highly significant and strong positive correlation. This may be related to the cation–anion exchange process in the water. The correlation between Ca^2+^ and Mg^2+^ is 0.492, possibly associated with the dissolution of carbonate rocks. Additionally, significant positive correlations are observed between pH and both Mg^2+^ (0.582) and F^−^ (0.460), indicating that Mg^2+^ and F^−^ may influence the pH of the water.

#### Principal component analysis

**Fig. 4:**
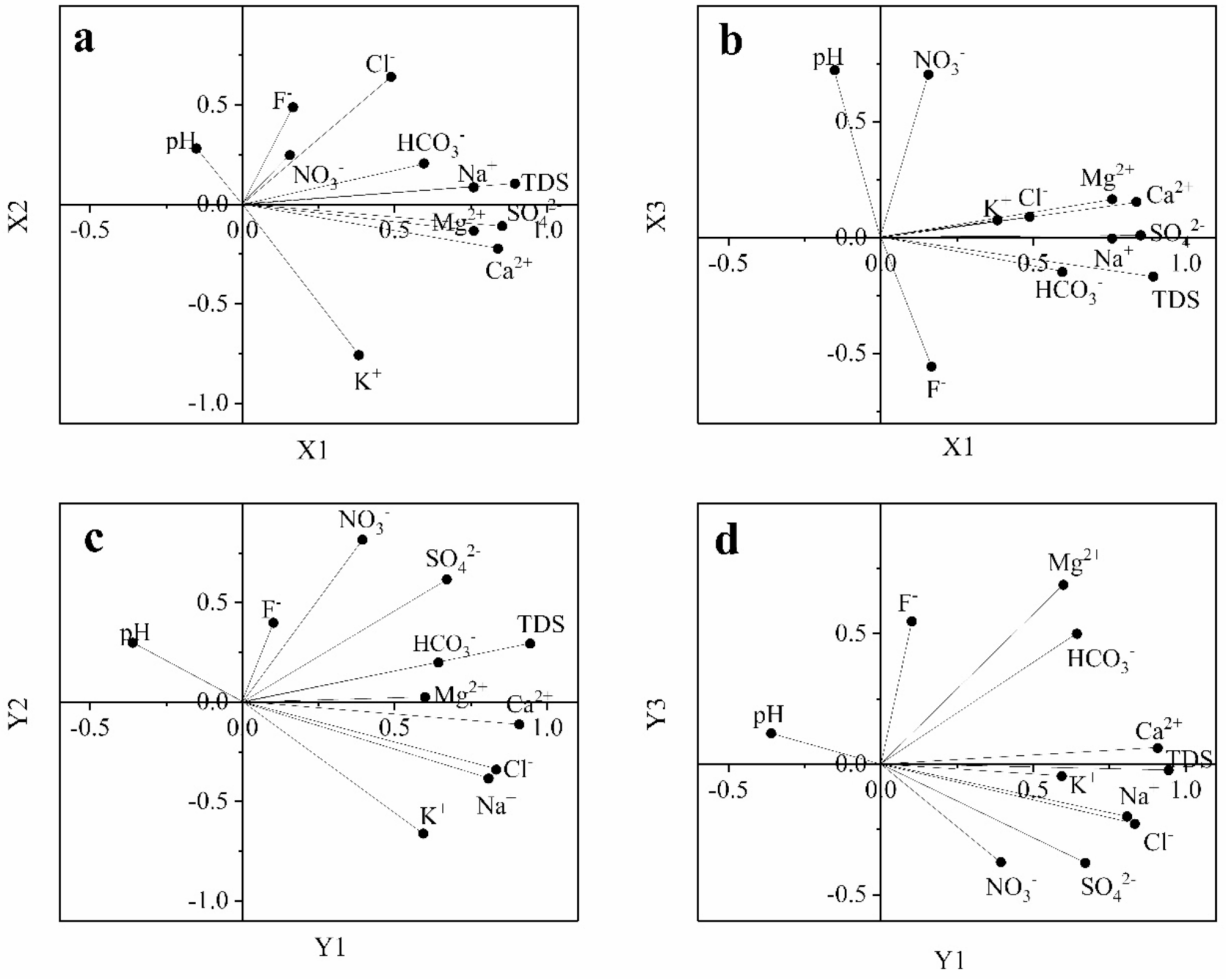
PCA loading plots of ions: (**a**) PCA loading plots for X1 and X2 in groundwater; (**b**) PCA loading plots for X1 and X3 in groundwater; (**c**) PCA loading plots for Y1 and Y2 in surface water; (**d**) PCA loading plots for Y1 and Y2 in surface water.

PCA based on key chemical indices in water can effectively reflect the relationships between ionic compositions and the controlling factors influencing water quality^38–40^. In this study, eleven indicators of water samples from the area were subjected to PCA, using the criterion that eigenvalue exceed one. Four principal component factors influencing water quality evolution were identified. The cumulative variance contribution rates for groundwater and surface water are 75.86% and 87.64%, respectively. The results of the principal component factor extraction for groundwater and surface water are presented in Table 3. The factor loading plots based on the principal component factor scores are shown in Fig. 4. The straight line connecting each variable to the origin indicates the contribution of that variable to the sample. The proximity of the straight lines representing two variables reflects the strength of their interconnection^41–43^.

**Table 3:** Loading matrix of principal component factors.

Variable	Groundwater				Surface water			
	X1	X2	X3	X4	Y1	Y2	Y3	Y4
K^+^	0.38	− 0.76	0.07	0.18	0.59	− 0.66	− 0.05	0.04
Na^+^	0.76	0.09	− 0.004	0.03	0.81	− 0.39	− 0.20	0.29
Ca^2+^	0.84	− 0.22	0.15	0.10	0.91	− 0.11	0.06	0.25
Mg^2+^	0.76	− 0.13	0.17	− 0.34	0.60	0.03	0.69	0.01
HCO_3_^−^	0.60	0.21	− 0.15	− 0.67	0.64	0.20	0.50	− 0.48
Cl^−^	0.49	0.64	0.09	− 0.17	0.83	− 0.34	− 0.23	0.18
SO_4_^2−^	0.85	− 0.11	0.01	0.34	0.67	0.62	− 0.38	0.03
F^−^	0.17	0.49	− 0.56	0.54	0.10	0.40	0.55	0.37
NO_3_^−^	0.16	0.25	0.70	0.07	0.39	0.82	− 0.38	− 0.07
TDS	0.89	0.11	− 0.17	0.25	0.94	0.30	− 0.02	− 0.10
pH	− 0.15	0.28	0.72	0.32	− 0.36	0.30	0.12	0.78
Variance %	38.13	13.69	13.07	10.96	44.87	19.40	12.89	10.48
Cumulative variance %	38.13	51.82	64.89	75.86	44.87	64.27	77.16	87.64

Based on the magnitude of the factor loading values, the results of PCA were categorized into five types: “very high positive” (> 0.9), “high positive” (0.75–0.90), “medium positive” (0.60–0.75), “low positive” (0.45–0.60), and “very low positive” (< 0.45)^42–45^. A total of four principal component factors were extracted from the groundwater, accounting for 75.86% of the cumulative variance. Factor X1 explained 38.13% of the total variance. Na^+^, Mg^2+^, Ca^2+^, SO_4_^2−^, and TDS exhibited high positive loadings (Table 3, Fig. 4a), and their near-parallel alignment with X1 in Fig. 4a indicates their strong contribution to this factor. Factor X2 contributed 13.69% of the variance, with Cl^−^ showing medium positive loading values and K^+^ displaying high negative loading values (Table 3, Fig. 4a). Factor X3 explained 13.07% of the total variance, with NO_3_^−^ and pH showing medium positive loadings, indicating their significant contribution to X3 (Table 3, Fig. 4b). Factor X4 accounted for 10.96% of the variance, with F^−^ exhibiting low positive loading values and HCO_3_^−^ showing a high negative loading. This may be related to the greater stability of F^−^ in alkaline water environments^46^.

A total of four principal component factors were extracted from surface water, contributing to 87.64% of the cumulative variance. Factor Y1 accounted for 44.87% of the variance. Mg^2+^ and HCO_3_^−^ exhibited medium positive loadings, Na^+^ and Cl^−^ showed high positive loadings, and TDS and Ca^2+^ had very high positive loading values. This indicates that Na^+^, Cl^−^, TDS, and Ca^2+^ made the primary contributions to Y1 (Table 3, Fig. 4c). Factor Y2 explained 19.4% of the total variance, with SO_4_^2−^ and NO_3_^−^ showing medium to high positive loading values, and K^+^ exhibiting a high loading in Y2 (Table 3, Fig. 4c). F^−^ displayed a low positive loading, while Mg^2+^ showed a medium positive loading in factor Y3, which explained 12.89% of the total variance (Table 3, Fig. 4d). Factor Y4 contributed 10.48% of the variance, with pH showing high positive loadings, making a significant contribution to Y4.

It can be observed that the first principal component factor (X1) of groundwater is primarily associated with the SO_4_·HCO_3_-Ca·Na type, as well as some HCO_3_·SO_4_-Ca·Na and HCO_3_-Ca·Mg(II) types of groundwater (Supplementary Fig. S3a, Supplementary Fig. S2). It is speculated that X1 may be influenced by the weathering and dissolution of silicate rocks, as well as the weathering and sulfate dissolution of carbonate rocks. The principal component factor X2 is primarily associated with the SO_4_·HCO_3_-Ca·Na type of groundwater (Fig. S3a). Since water samples of this type are distributed downstream of human settlements (Fig. S2), it suggests that X2 may be influenced by domestic wastewater. The principal component factor X3, which shows medium positive loadings for NO_3_^−^ and pH, is influenced by the physicochemical properties of the water (Fig. S3b). The decrease in pH is attributed to the hydrolysis of acidic substances^47^. In agricultural areas, the use of inorganic fertilizers^48,49^ leads to NO_3_^−^ pollution, indicating that X3 reflects the impact of agricultural wastewater.

The Y1 factor of surface water is primarily associated with the SO_4_·HCO_3_-Ca and SO_4_·HCO_3_-Ca·Na types, as well as a small amount of HCO_3_-Ca·Na and HCO_3_-Ca·Mg types of surface water (Fig. S3c, Fig. S2). It is speculated that principal component factor Y1 may be influenced by silicate weathering and human activities. The Y2 factor is primarily associated with the SO_4_·HCO_3_-Ca type and some HCO_3_-Ca·Mg type of surface water (Fig. S3c, Fig. S2), suggesting that Y2 may be influenced by industrial activities. The Y3 factor is mainly associated with the HCO_3_-Ca·Mg type and some HCO_3_-Ca·Na type of surface water (Fig. S3d). The water samples of the HCO_3_-Ca·Mg type are predominantly found in carbonate areas, indicating that Y3 may be influenced by carbonate weathering.

### Analysis of major ion sources and control factors

#### Water–rock model analysis

**Fig. 5:**
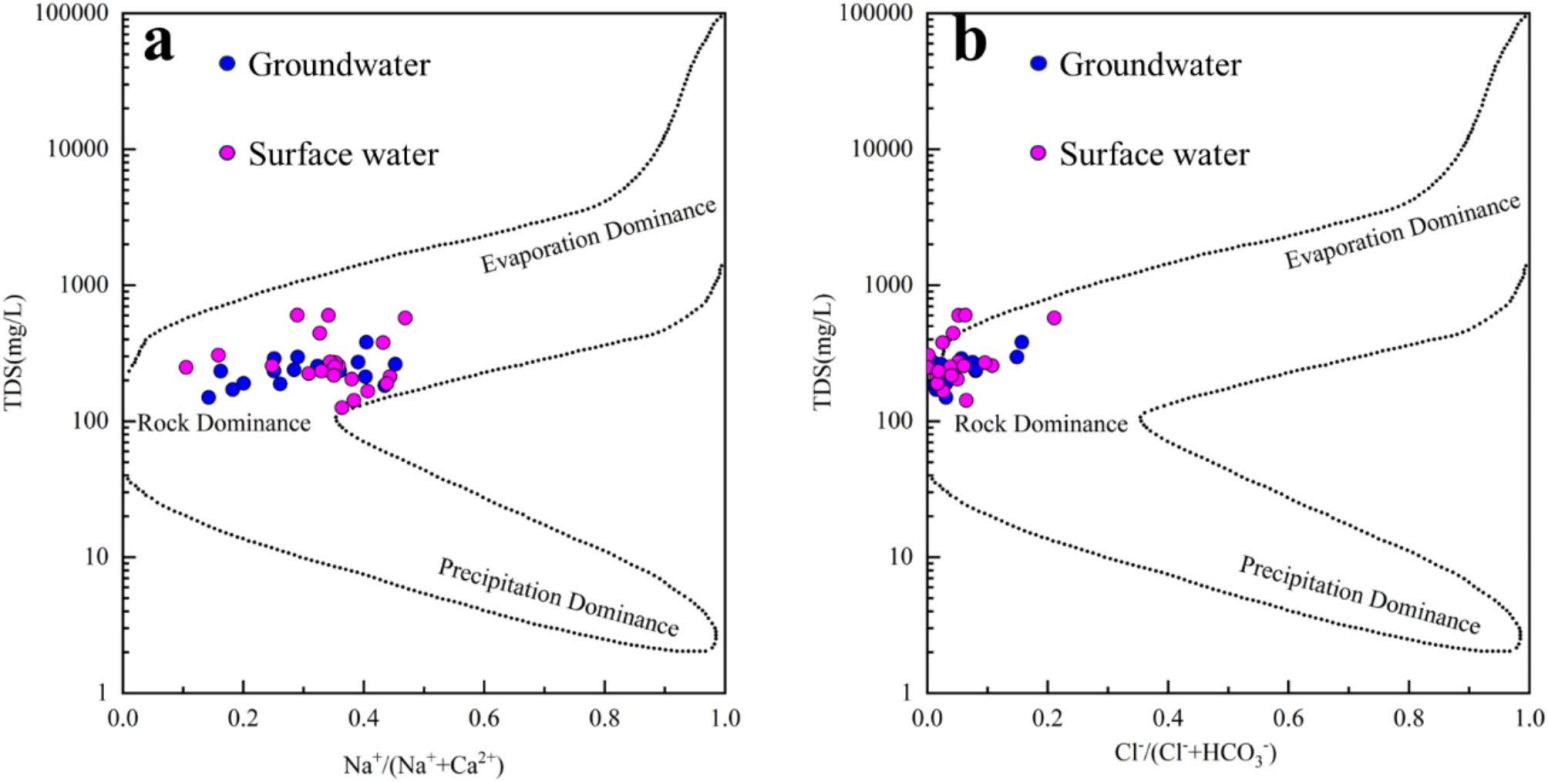
Water–rock interaction model: Gibbs diagram of water chemistry.

Gibbs developed a semi-logarithmic plot where the ordinate represents TDS as a logarithmic value, and the abscissa represents either [Na^+^: (Na^+^ + Ca^2+^)] or [Cl^−^: (Cl^−^ +  HCO_3_^−^)]. This plot provides a direct reflection of the effects of evaporation/crystallization, rock weathering, and atmospheric precipitation on groundwater and surface water^50^. Most of the water samples in the study area fall in the middle and tend toward the left side of the Gibbs diagram (Fig. 5). This region corresponds to the rock-dominated domain, and all samples are located far from the precipitation domain, indicating that the hydrochemical ion composition in the study area is primarily influenced by rock weathering.

Atmospheric precipitation is typically considered the baseline, and a logarithmic relationship model is used between TDS and the ratio of (Ca^2+^: Na^+^) to study the factors affecting hydrochemistry^12^. Rainwater samples were collected from the basin, with TDS, Ca^2+^, and Na^+^ concentrations of 63.06, 14.88, and 2.44 mg/L, respectively. Based on this data, a relationship model between lg (Ca^2+^/Na^+^) and lg (TDS) was plotted (Supplementary Fig. S4). Most of the sampling points fall within the rock-dominated domain, and the results align with the earlier Gibbs model analysis. With the exception of a few surface water samples influenced by the weathering of Ca-containing rocks, the majority of the water samples are primarily affected by the weathering of Na-containing rocks.

**Fig. 6:**
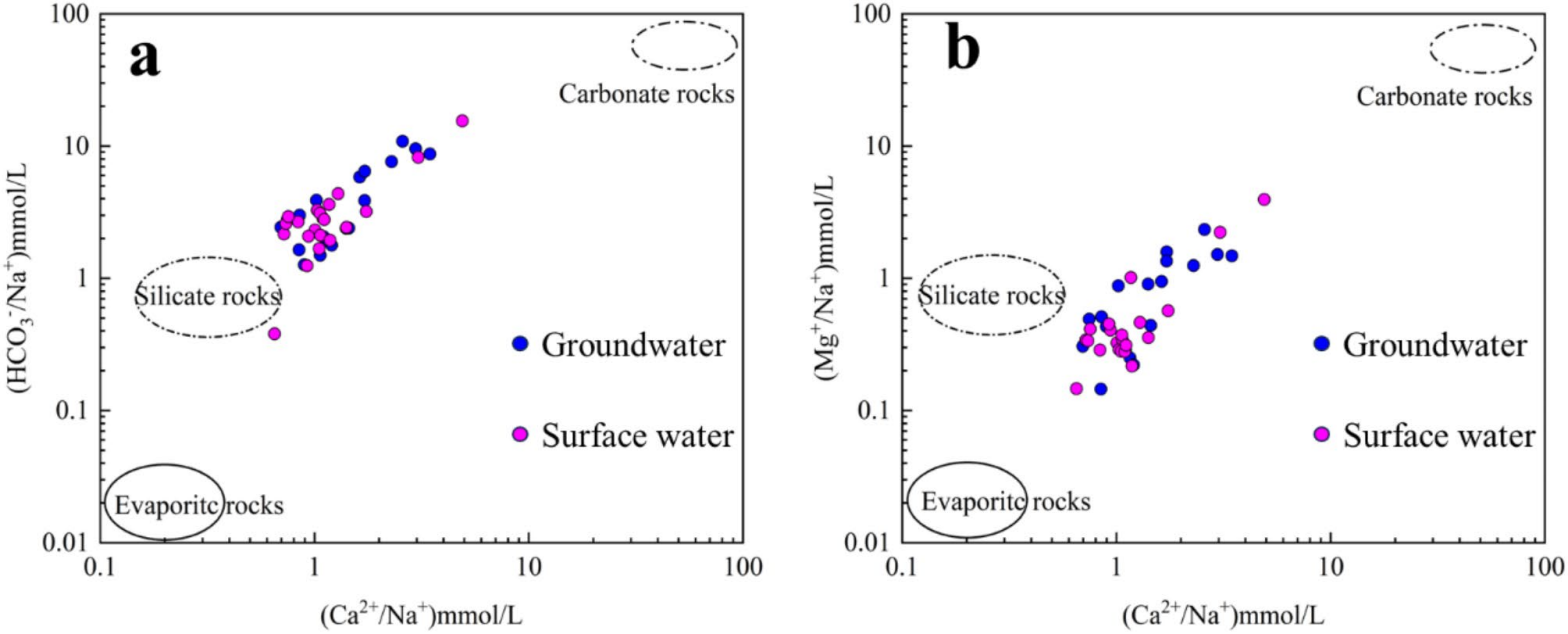
Water–rock interaction model: Diagrams of Na-normalized molar ratios.

The ratios of Ca^2+^/Na^+^, Mg^2+^/Na^+^, and HCO_3_^−^/Na^+^ (mmol/L) in water are not influenced by flow velocity, dilution, or evaporation, making them valuable for qualitatively identifying the effects of weathering on hydrochemical components in carbonate, silicate, and partial evaporite rocks^51,52^. The samples are concentrated in the middle of the silicate and carbonatite domains, with a stronger inclination toward the silicate domain (Fig. 6). This suggests that most of the groundwater and surface water in the study area are influenced by the dissolution of silicate and carbonatite minerals. It is speculated that this is related to the widespread distribution of Variscan diorite and carbonate rocks in the southeastern parts of the area.

#### Ion exchange

Ion exchange is a process in which, under certain conditions, granules adsorb specific cations from water and release previously adsorbed cations back into the water^53^. The molar concentration ratio of (Na^+^–Cl^−^) to [(2SO_4_^2−^ +  HCO_3_^−^)–2(Ca^2+^ + Mg^2+^)] (mmol/L) serves as an indicator of ion exchange processes involving sodium and chloride ions^54^. If the hydrochemical evolution process is primarily governed by ion exchange, the ratio of (Na^+^– Cl^−^) to [(2SO_4_^2−^ + HCO_3_^−^)–2(Ca^2+^ + Mg^2+^)] should be distributed around the 1:1 line. Some of the ratios in water samples are near the 1:1 line (Supplementary Fig. S5), while others deviate significantly from it. These results suggest that ion exchange influences the hydrochemical evolution process in certain parts of the area.

#### Main weathering processes and water chemical evolution

**Fig. 7:**
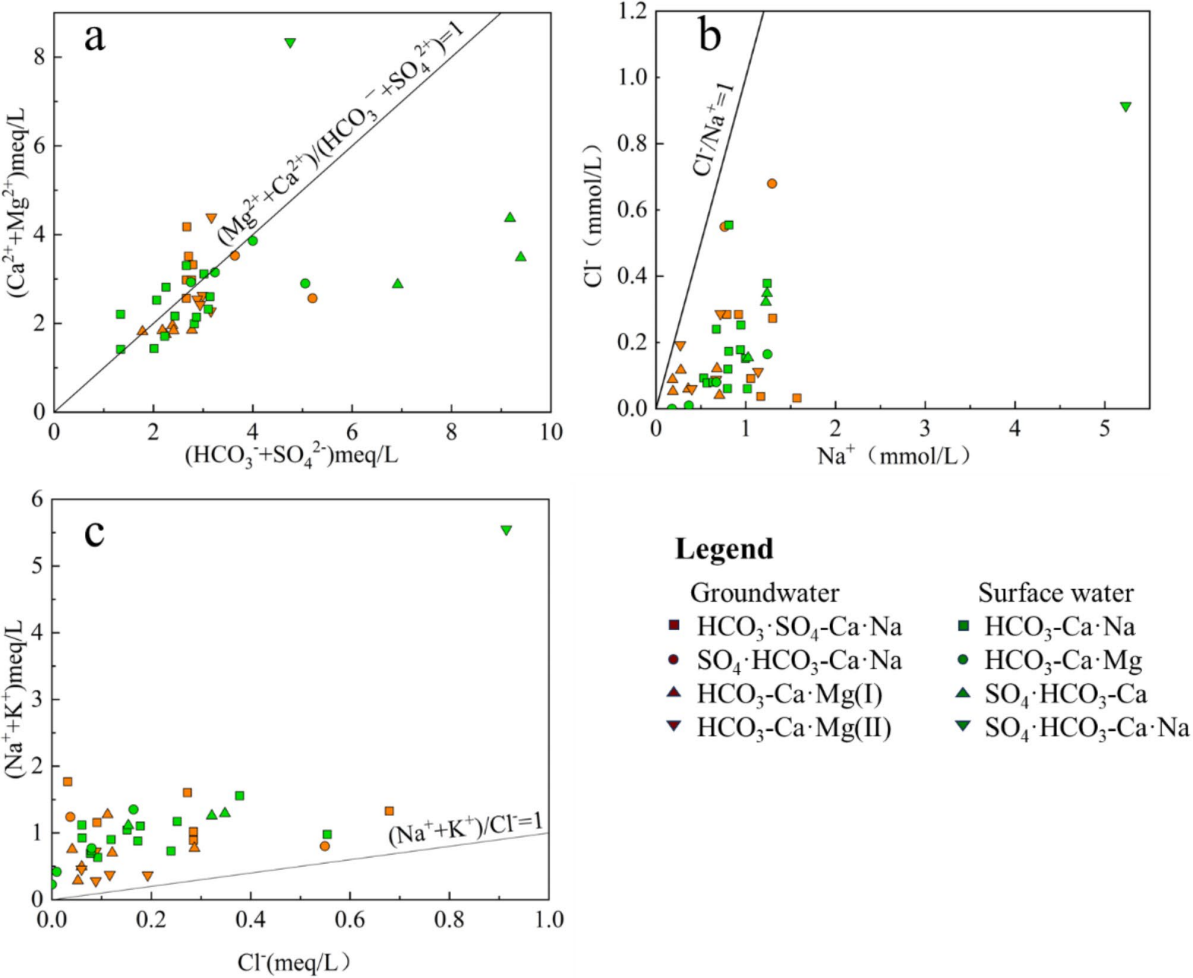
Ion concentration ratios: (**a**) (Ca^2+^ + Mg^2+^) versus (HCO_3_^−^ + SO_4_^2−^); (**b**) Cl^−^ versus Na^+^; (**c**) (Na^+^ + K^+^) versus Cl^−^.

The primary source of Mg^2+^ and Ca^2+^ ions in the local water is the dissolution of magnesium- and calcium-bearing silicates and carbonates. The origin of these ions can be inferred from the scatter plot of the [(Mg^2+^ + Ca^2+^) : (HCO_3_^−^ + SO_4_^2−^)] ratio^55–58^. In the scatter diagram of (Mg^2+^ + Ca^2+^) versus (HCO_3_^−^ + SO_4_^2−^), data points above the equiline indicate carbonate weathering, while points along the equiline suggest a combination of both carbonate and silicate weathering^55^. The majority of sample data from the area cluster around to the equiline (Fig. 7a), suggesting that both carbonate and silicate weathering are the primary sources of Ca^2+^ and Mg^2+^ ions in the water. However, the SO_4_·HCO_3_-Ca sample in surface water deviates significantly from the equiline. Cluster analysis reveals that this sample type is predominantly found downstream, in an area characterized by groundwater discharge. Upstream human activities have led to elevated SO_4_^2−^ levels, resulting in an increased (HCO_3_^−^ + SO_4_^2−^) value. Consequently, the ratio is positioned below and away from the equiline.

Na ions in the water are primarily derived from the dissolution of salt rock, which releases equal concentrations of Na^+^ and Cl^−^^59^. However, the Na^+^: Cl^−^ ratio in the water samples from the area deviates significantly from the equiline, instead clustering near the Na^+^ axis (Fig. 7b). This discrepancy indicates that the concentration of Na^+^ exceeds that of Cl^−^, suggesting the presence of additional sodium sources beyond salt rock dissolution.

The ratio of (Na^+^ + K^+^) to Cl^−^ can be employed to identify the primary source of Na^+^ and K^+^ in the water. A ratio of 1 indicates salt rock dissolution, whereas a ratio greater than 1 suggests silicate rock weathering^5,60^. Notably, all sampling points in the study area are positioned above the salt rock dissolution line (Fig. 7c), indicating that the water samples have undergone significant silicate weathering. This finding, combined with the results of the lg (Ca^2+^/Na^+^) and lg (TDS) relationship models, and the widespread presence of diorite in the area, suggests that diorite weathering is the primary influence on Na^+^ and K^+^ concentrations in the water.

Correlation analysis reveals a significant correlation between Na^+^, K^+^, and Cl^−^ in surface water. Notably, despite this correlation, the absence of significant evaporite outcrops in the area suggests that silicate weathering alone cannot account for the Na^+^ and K^+^ concentrations in surface water. Instead, these findings imply that additional sources, such as human activities, contribute to the elevated levels of Na^+^ and K^+^ in surface water.

#### Human activity impact

Cluster and principal component analyses indicate that the hydrochemical characteristics of HCO_3_-Ca·Na-type surface water are complex and heterogeneous. This water type exhibits significant variability in concentrations of K^+^, Na^+^, Cl^−^, and NO_3_^−^. Notably, HCO_3_-Ca·Na-type surface water is widespread, particularly in areas with high population densities and intensive agricultural activities. The use of synthetic fertilizers, such as animal manure, urea, and organic amendments, can increase concentrations of K^+^, Na^+^, Cl^−^, and NO_3_^−^ in water. However, the concentrations of these ions are generally lower in river valleys located farther from residential and agricultural areas, indicating reduced influence from human activities. Conversely, the concentrations of K^+^, Na^+^, Cl^−^, and NO_3_^−^ in surface water are mainly controlled by anthropogenic sources, including agricultural fertilizers, domestic wastewater, and livestock manure.

**Fig. 8:**
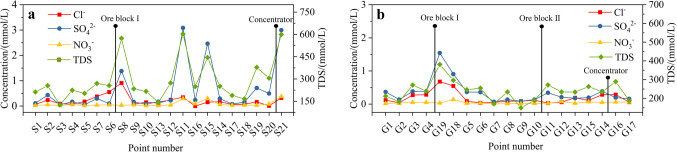
Spatial variation of SO_4_^2−^, NO_3_^−^, Cl^−^, and TDS concentrations in (**a**) surface water and (**b**) groundwater along the Jinzi River profile.

Pollutants from anthropogenic activities are typically enriched with K^+^, Ca^2+^, SO_4_^2−^, Cl^−^, and NO_3_^−^ though K^+^, Ca^2+^, SO_4_^2−^, and Cl^−^ can also result from rock weathering. Consequently, variations in NO_3_^−^ levels serve as key indicators of human impact on the water chemistry^61,62^. SO_4_^2−^ in rivers is generally attributed to the oxidation of sulfides, industrial emissions of SO_2_ from activities such as coal combustion, and subsequent atmospheric deposition^63^. The data indicate that groundwater runoff is steady in the mountainous areas and along the slopes flanking the Jinzi River. The groundwater in these regions is characterized by low-mineralized freshwater. However, the chemical composition evolves due to the influence of the host rock composition and the presence of sulfide-type gold ore bodies, leading to a gradual increase in sulfate ions in the valley and surrounding water. Consequently, NO_3_^−^, SO_4_^2−^, Cl^−^, and TDS are used as indicators to assess the impact of human activities on hydrochemistry. As shown in Fig. 8b, the trends of SO_4_^2−^, NO_3_^−^, Cl^−^, and TDS in groundwater across the area are largely consistent. A sharp increase in sulfate ions is observed near the mining area, contributing to deteriorating water quality and a relative rise in the concentrations of other indicators. Similarly, the trends of SO_4_^2−^, NO_3_^−^, Cl^−^, and TDS in surface water exhibit a comparable pattern (Fig. 8a). There is a notable increase in concentrations near the discharge outlets of the sewage plant. However, as the water progresses downstream, undergoing self-purification and dilution through the contribution of tributaries, these concentrations gradually decrease. This process mitigates the impact of mineral extraction activities on the hydrochemistry of the basin. Nonetheless, it is evident that upstream human activities exert a significant influence on the hydrochemistry of the Jinzi River Basin.

People often use the ratio of SO_4_^2−^/Ca^2^⁺ and NO_3_⁻/Ca^2^⁺ to analyze the impact of human activities on the major ions in water. When SO_4_^2−^/Ca^2^⁺ > NO_3_⁻/Ca^2^⁺, it indicates that human activities are more influenced by industrial and mining activities; conversely, when SO_4_^2−^/Ca^2^⁺ < NO_3_⁻/Ca^2^⁺, it suggests that agricultural activities and domestic wastewater have a greater impact^22,64^. In the area, most groundwater samples are located at the industrial and mining activity end-member, while surface water samples are at the agricultural activity and domestic wastewater end-member (Supplementary Fig. S6a). This indicates that groundwater is mainly influenced by industrial and mining activities, while surface water is primarily affected by agricultural activities and domestic wastewater.

Cl⁻ is a conservative element in natural water environments. Higher concentrations of Cl⁻ in water may indicate the presence of various anthropogenic sources in the area. The content of Cl⁻ in domestic wastewater is relatively high, and it has stable chemical properties. Comparing the concentrations of Cl⁻ and NO_3_⁻ in the water can provide insights into the sources of NO_3_⁻^34,65^. To further investigate the sources of NO_3_⁻ in surface water, the water samples from the Supplementary Fig. S6a that were located at agricultural activities and domestic wastewater were plotted on a triangular diagram (Fig. S6b). The surface water samples are closer to the fertilizer side, indicating that the use of fertilizers in agricultural activities has a significant impact on surface water. Meanwhile, domestic wastewater and manure are also potential sources of nitrate in the surface water of the area. Most of the groundwater samples collected in the region are distributed around the fertilizer and manure end-members, suggesting that agricultural activities are also an important source of nitrate in the area.

### Discussion

This study analyzed the hydrochemical characteristics and influencing factors of surface water and groundwater in the Jinzi River Basin. Water samples were collected and subjected to comprehensive analysis using multivariate statistical tools, including the Piper trilinear diagram, Gibbs model, ionic correlation, and ionic ratio relationships. These methods facilitated the identification of hydrochemical characteristics and an in-depth discussion of the factors influencing them. The water in the Jinzi River Basin is weakly alkaline, with the primary hydrochemical type for groundwater being HCO_3_-Ca·Mg and for surface water being HCO_3_-Ca·Na. These findings align with previous research in the area^66,67^. Unlike many existing studies that broadly address hydrochemical characteristics^14,68,69^, this study delves deeper by employing cluster analysis to specifically classify and analyze the various hydrochemical types in the region. Additionally, the study revealed that the same hydrochemical type exhibits varying component concentrations due to differences in rock weathering processes across the region. Unlike other scholars^31–34^ who primarily used cluster analysis to identify the influencing factors of watershed hydrochemistry, this research uniquely correlated the distribution of CA results with specific hydrochemical types. This approach allowed for a more detailed identification of the factors influencing hydrochemical characteristics in different areas.

The study confirms that water–rock interactions and human activities are the key factors shaping the hydrochemical composition, aligning with previous research findings^22,31^. The results of both PCA and cluster analysis support this conclusion. Groundwater and surface water in the area are notably influenced by water–rock interaction, alongside significant contributions from anthropogenic sources such as industrial activities, agricultural practices, and domestic wastewater discharge. The findings highlight the effectiveness of multivariate statistical methods, such as PCA and cluster analysis, in investigating hydrochemical characteristics and influencing factors in this region^18,68^. These methods, supported by the Gibbs model and ion ratio analyses, revealed that Ca^2+^, Mg^2+^, and HCO_3_^−^ primarily originate from the weathering and dissolution of diorite, with additional contributions from carbonate rock weathering. Conversely, SO_4_^2−^ and NO_3_^−^ concentrations were significantly influenced by human activities, while Na^+^, K^+^, and Cl^−^ were mainly attributed to the weathering and dissolution of silicate rocks, including diorite. The study also observed that certain ions, including Na^+^, K^+^, and Cl^−^ were significantly influenced by human activities, such as industrial, agricultural, and domestic wastewater discharge. Moreover, the consistent spatial variation in the concentrations of SO_4_^2−^, NO_3_^−^, Cl^−^, and TDS along the Jinzi River profile suggests a significant influence of human activities on the water chemistry.

The hydrochemical composition of groundwater and surface water in the area is influenced by a combination of geological and geomorphological conditions, as well as natural hydroclimatic processes and anthropogenic activities. The study found that natural factors, particularly the weathering and dissolution of local rocks, predominantly control the formation of major hydrochemical indicators. The increasing influence of human activities, including industrial, agricultural, and domestic wastewater discharge, is becoming more evident, particularly in the concentrations of ions like NO_3_^−^, SO_4_^2−^, and Cl^−^. Understanding the geochemical processes that control the chemical composition of surface and groundwater is essential for developing effective water management strategies. Therefore, it is essential that relevant authorities strengthen water quality monitoring and management practices to ensure the scientific management and sustainable utilization of water resources within the Jinzi River Basin.

## Conclusion

In the current study, the chemical parameters of 40 surface water and groundwater samples from the area were analyzed, including major ions, TDS, pH, and other relevant indicators. The main findings of this study are as follows:

1. The primary cations in groundwater were Ca^2+^ and Mg^2+^, with HCO_3_^−^ as the dominant anion, resulting in a hydrochemical type primarily characterized as HCO_3_-Ca·Mg. In surface water, the main cations were Ca^2+^ and Na^+^, with HCO_3_^−^ as the predominant anion, leading to a hydrochemical type mainly characterized as HCO_3_-Ca·Na.
2. The results of the cluster analysis indicated that the primary hydrochemical components in the area were influenced by the weathering of silicate and carbonate rocks. The same hydrochemical type, influenced by the weathering of different rocks, showed varying concentrations of each component. Notably, the concentrations of component in HCO_3_-Ca·Mg water from the carbonate-rich area were significantly higher than those in the silicate-rich area.
3. The results of the PCA suggested that the main hydrochemical components in the area were influenced by both rock weathering and human activities. The X1 factor in groundwater was primarily influenced by the dissolution from rock weathering, while the X2 factor was affected by domestic wastewater. The X3 factor was impacted by agricultural wastewater. Additionally, the Y1 factor was influenced by rock weathering, Y2 by industrial activities, and Y3 by the weathering of carbonate rocks.
4. The Gibbs model and the Ca-Na-TDS relationship model collectively indicated that rock weathering was the primary controlling factor for the main ions in the water. The ratio relationships between Ca^2+^/Na^+^, HCO_3_^−^/Na^+^, and Mg^2+^/Na^+^ further confirmed that the water chemistry was influenced by the weathering of both silicate and carbonate rocks. Additionally, the ratio relationship between (Na^+^– Cl^−^)/[(2SO_4_^2−^ + HCO_3_^−^)–2(Ca^2+^ + Mg^2+^)] suggested the occurrence of cation exchange in this area.
5. The ion ratio analysis indicated that Ca^2+^, Mg^2+^, and HCO_3_^−^ primarily originated from the weathering and dissolution of diorite, with partial contributions from carbonate rocks. SO_4_^2−^ and NO_3_^−^ were mainly influenced by human activities. Na^+^, K^+^, and Cl^−^ primarily originated from the weathering and dissolution of silicate rocks, such as diorite, with significant contributions from human activities, including industrial, agricultural, and domestic wastewater discharge.
6. The spatial variation characteristics of SO_4_^2−^, NO_3_^−^, Cl^−^, and TDS concentrations along the Jinzi River profile showed consistent patterns, indicating a significant influence of human activities.

## Electronic supplementary material

Below is the link to the electronic supplementary material.

Supplementary Material 1

## Data Availability

All data generated or analysed during this study are included in this published article and its Supplementary Information files.
