# Supplementary material for: Chemical characteristics of groundwater and surface water affected by human activities in the upper Jinzi River Basin, China
Source: Sci Rep. 2025 Mar 18;15:9294. doi: 10.1038/s41598-025-93318-5 (PMC11920243; doi:10.1038/s41598-025-93318-5)
Supplement: Supplementary file 1 — Supplementary Material 1 [file 41598_2025_93318_MOESM1_ESM.docx]

**Chemical Characteristics of Groundwater and Surface Water under the Influence of Human Activities in the Upper Jinzi River Basin,China**

Xiaochen Zhang ^1,2,3,4^, Yongcheng Zhang ^1,2,3,4^, Yingjia Gui ^1,2,3,4^, Ruiyan Sun ^1,2,3,4^, Jun Li ^1,2,3,4^, Qi Wu ^1,2,3,4^, Yongkang Ding ^5^, Kang Chen ^1,2,3,4,^*

1. School of Water Resources and Environment, Hebei GEO University, Shijiazhuang **050031, China**

2. Hebei Province Key Laboratory of Sustained Utilization and Development of Water Resources, Hebei GEO University, Shijiazhuang, **050031, China**

3. Hebei Province Collaborative Innovation Center for Sustainable Utilization of Water Resources and Optimization of Industrial Structure, Hebei GEO University, Shijiazhuang, **050031, China**

4. Hebei Center for Ecological and Environmental Geology Research, Hebei GEO University, Shijiazhuang, **050031, China**

5. Xi’an Center of Mineral Resources Survey, China Geological Survey, Xi’an, **710100, China**

**Supplementary Table S1** Statistics on the characteristics of various types of groundwater and surface water physical and chemical indicators (average)/(mg/L)

| indicators | | K^+^ | Na^+^ | Ca^2+^ | Mg^2+^ | HCO_3_^-^ | Cl^-^ | SO_4_^2-^ | F^-^ | NO_3_^-^ | TDS | pH |
| --- | --- | --- | --- | --- | --- | --- | --- | --- | --- | --- | --- | --- |
| Ground-water | HCO_3_·SO_4_-Ca·Na | 5.65 | 26.08 | 50.51 | 8.75 | 120.2 | 5.93 | 35.66 | 0.07 | 2.58 | 252.77 | 7.56 |
|  | SO_4_·HCO_3_-Ca·Na | 1.39 | 23.66 | 43.39 | 10.53 | 120.4 | 21.81 | 117.5 | 0.14 | 5.36 | 338.67 | 7.49 |
|  | HCO_3_-Ca·Mg(I) | 3.01 | 9.17 | 22.94 | 8.35 | 120.44 | 2.84 | 15.41 | 0.07 | 1.68 | 182.15 | 7.33 |
|  | HCO_3_-Ca·Mg(II) | 3.45 | 14.69 | 33.53 | 14.13 | 162.18 | 5.254 | 17.64 | 0.07 | 3.116 | 250.87 | 7.37 |
| Surface water | HCO_3_-Ca·Na | 5.06 | 19.06 | 33.9 | 7.11 | 125.98 | 6.60 | 16.46 | 0.06 | 25.2 | 214.17 | 7.36 |
|  | HCO_3_-Ca·Mg | 3.05 | 14.12 | 37.11 | 16.27 | 180.46 | 2.26 | 38.58 | 0.09 | 3.86 | 291.84 | 7.11 |
|  | SO_4_·HCO_3_-Ca | 2.25 | 26.74 | 57.56 | 8.36 | 170.63 | 9.75 | 273.54 | 0.07 | 20.09 | 548.83 | 7.45 |
|  | SO_4_·HCO_3_-Ca·Na | 12.36 | 120.35 | 136.27 | 18.36 | 121.41 | 32.46 | 132.62 | 0.07 | 2.17 | 573.83 | 7.52 |

**Supplementary Table S2** Correlation coefficient matrix of groundwater and surface water chemical parameters

|  |  | K^+^ | Na^+^ | Ca^2+^ | Mg^2+^ | HCO_3_^-^ | Cl^-^ | SO_4_^2-^ | F^-^ | NO_3_^-^ | TDS | pH |
| --- | --- | --- | --- | --- | --- | --- | --- | --- | --- | --- | --- | --- |
| Groundwater | K^+^ | 1 |  |  |  |  |  |  |  |  |  |  |
|  | Na^+^ | 0.367 | 1 |  |  |  |  |  |  |  |  |  |
|  | Ca^2+^ | 0.398 | 0.785^**^ | 1 |  |  |  |  |  |  |  |  |
|  | Mg^2+^ | -0.030 | -0.041 | 0.154 | 1 |  |  |  |  |  |  |  |
|  | HCO_3_^-^ | -0.107 | -0.053 | -0.094 | 0.455 | 1 |  |  |  |  |  |  |
|  | Cl^-^ | -0.184 | 0.275 | 0.347 | 0.026 | -0.110 | 1 |  |  |  |  |  |
|  | SO_4_^2-^ | -0.213 | 0.492^*^ | 0.401 | -0.163 | -0.207 | 0.869^**^ | 1 |  |  |  |  |
|  | F^-^ | -0.247 | 0.314 | 0.151 | -0.290 | -0.004 | 0.651^**^ | 0.835^**^ | 1 |  |  |  |
|  | NO_3_^-^ | -0.018 | 0.136 | 0.242 | 0.394 | 0.073 | 0.470^*^ | 0.317 | -0.056 | 1 |  |  |
|  | TDS | 0.010 | 0.705^**^ | 0.673^**^ | 0.195 | 0.247 | 0.748^**^ | 0.811^**^ | 0.654^**^ | 0.400 | 1 |  |
|  | pH | 0.050 | 0.163 | 0.413 | 0.203 | -0.157 | 0.196 | 0.160 | -0.114 | 0.461^*^ | 0.213 | 1 |
| Surface water | K^+^ | 1 |  |  |  |  |  |  |  |  |  |  |
|  | Na^+^ | 0.610** | 1 |  |  |  |  |  |  |  |  |  |
|  | Ca^2+^ | 0.471* | 0.911** | 1 |  |  |  |  |  |  |  |  |
|  | Mg^2+^ | 0.026 | 0.341 | 0.492* | 1 |  |  |  |  |  |  |  |
|  | HCO_3_^-^ | -0.202 | -0.052 | 0.128 | 0.370 | 1 |  |  |  |  |  |  |
|  | Cl^-^ | 0.641** | 0.839** | 0.829** | 0.152 | 0.060 | 1 |  |  |  |  |  |
|  | SO_4_^2-^ | -0.182 | 0.305 | 0.511* | 0.098 | 0.390 | 0.339 | 1 |  |  |  |  |
|  | F^-^ | -0.131 | -0.039 | 0.105 | 0.373 | 0.334 | -0.017 | 0.140 | 1 |  |  |  |
|  | NO_3_^-^ | -0.355 | 0.005 | 0.240 | -0.022 | 0.419 | 0.105 | 0.932** | 0.180 | 1 |  |  |
|  | TDS | 0.061 | 0.567** | 0.752** | 0.343 | 0.549** | 0.583** | 0.908** | 0.201 | 0.757** | 1 |  |
|  | pH | -0.020 | 0.026 | 0.205 | 0.582** | 0.257 | -0.080 | 0.091 | 0.460* | 0.059 | 0.182 | 1 |

Std: * and ** respectively represent at 0.05 and 0.01, the correlation is significant.


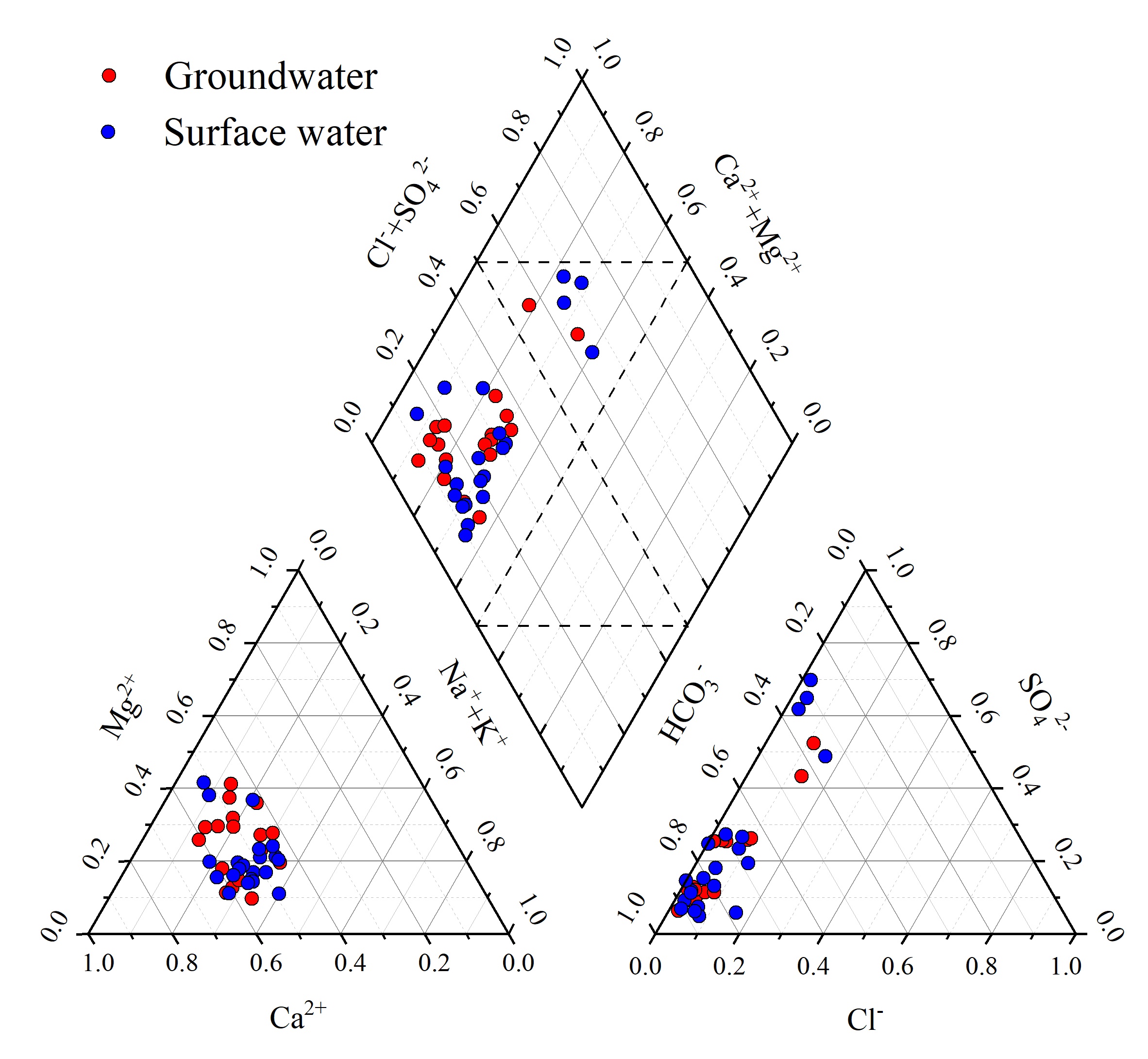


**Supplementary Fig. S1** Piper diagram for water samples in the area.


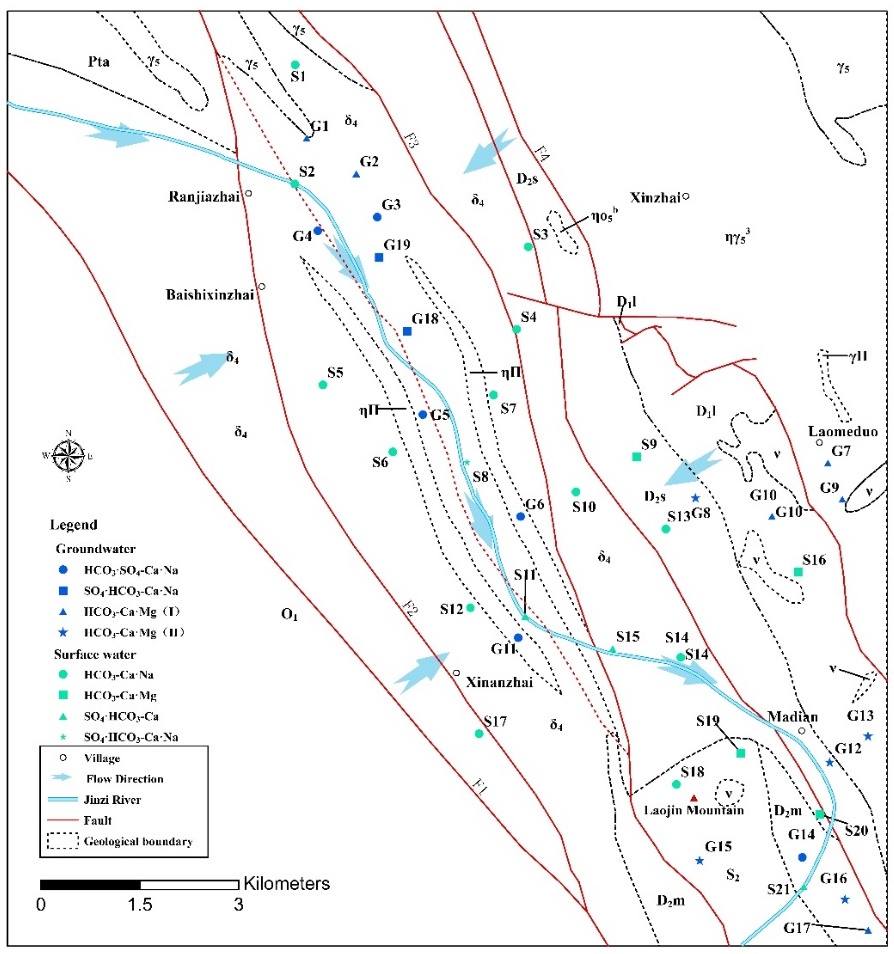


**Supplementary Fig. S2** Spatial distribution map of water sample points from cluster analysis results.


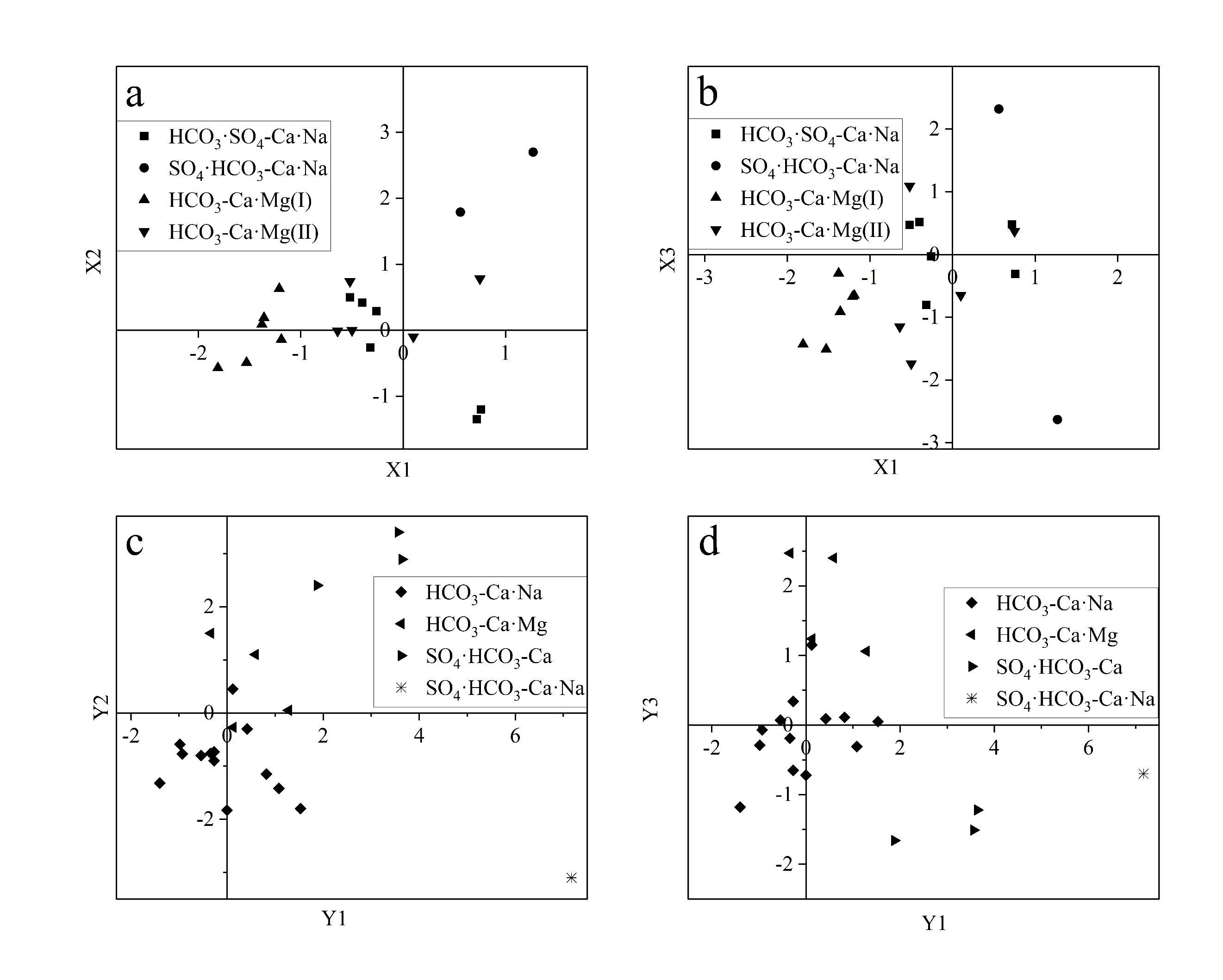


**Supplementary Fig. S3** Correlation diagram of PCA loading plots: **(a)** Correlation diagram of X1 and X2 in groundwater; **(b)** Correlation diagram of X1 and X3 in groundwater; **(c)** Correlation diagram of Y1 and Y2 in surface water; **(d)** Correlation diagram of Y1 and Y2 in surface water.


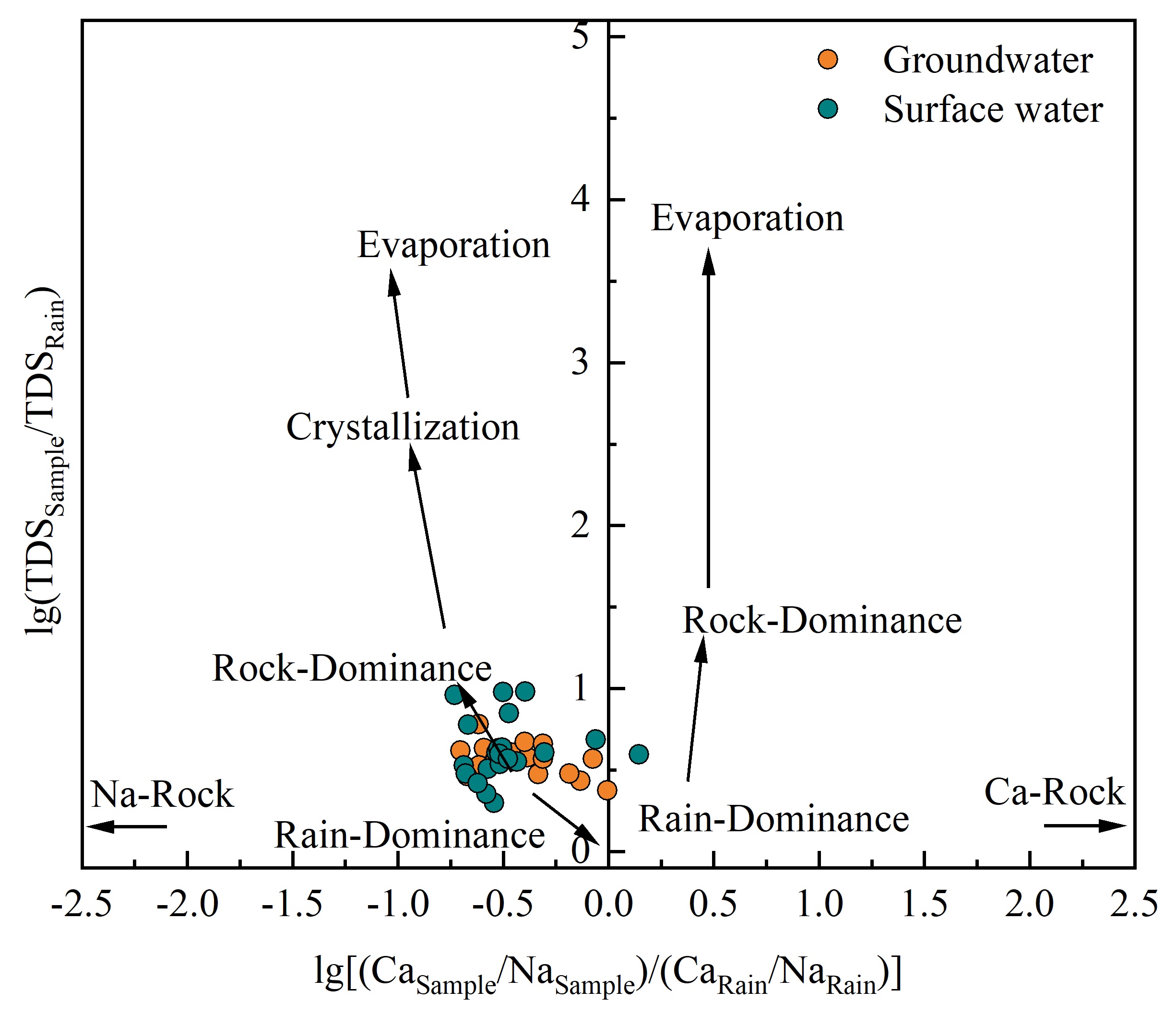


**Supplementary Fig. S4** Distribution map of Ca-Na-TDS model for the formation of hydrochemical compositions.


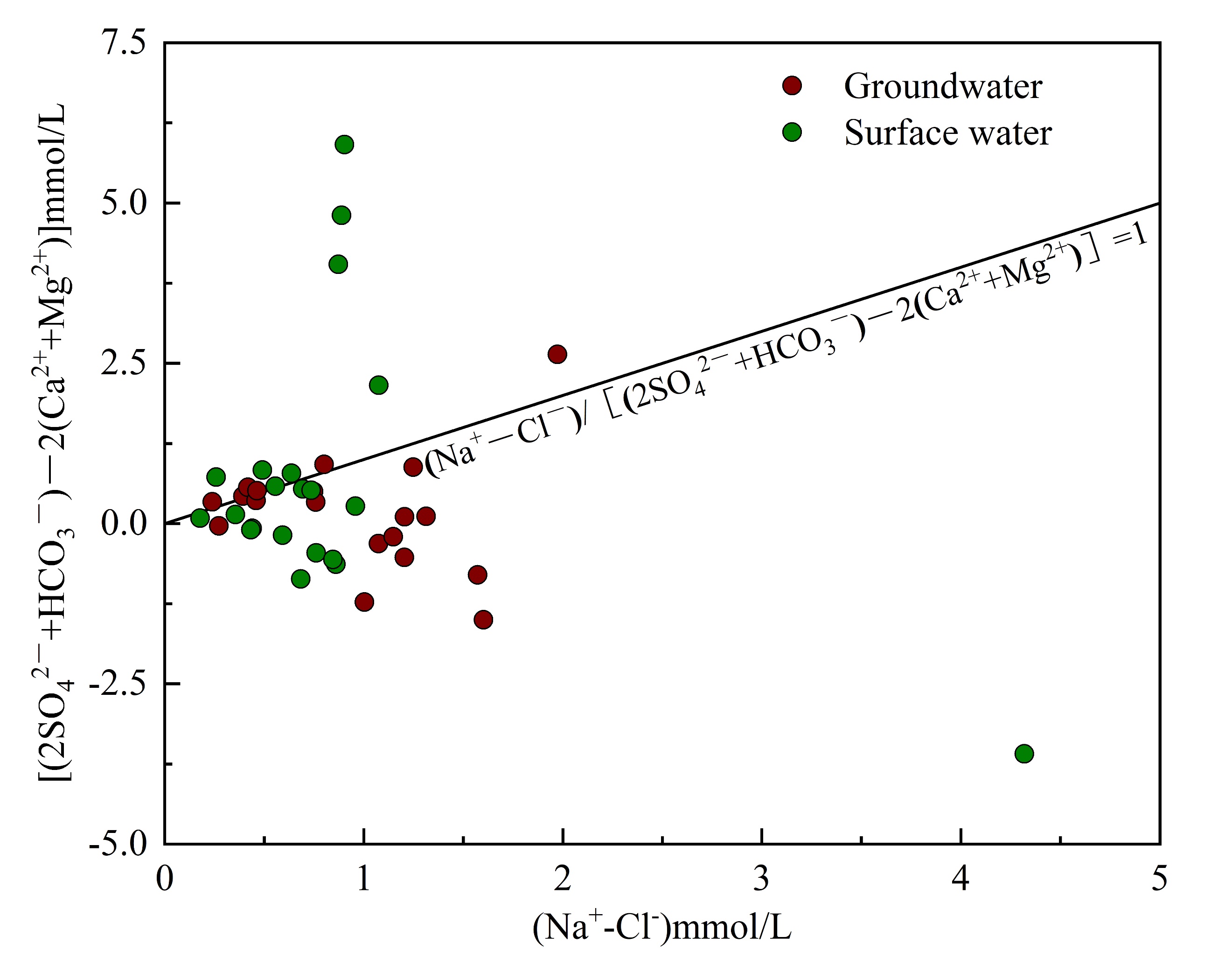


**Supplementary Fig. S5** (Na^+^-Cl^-^) versus [(2SO_4_^2-^+HCO_3_^-^)-2(Ca^2+^+Mg^2+^)].


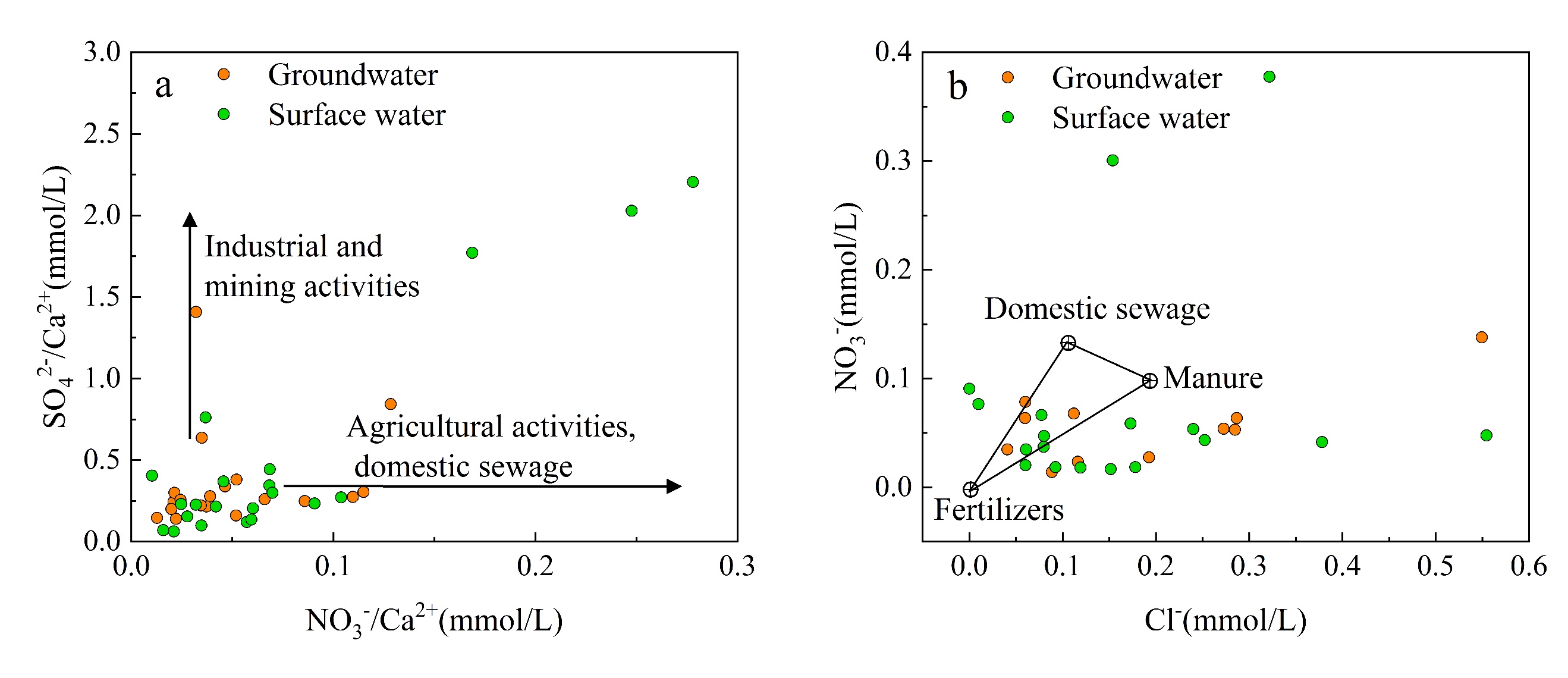


**Supplementary Fig. S6** The impact of human activities on ions: **(a)** (SO_4_^2+^/Ca^2+^) vs. (NO_3_^-^/Ca^2+^); **(b)** NO_3_^−^ VS. Cl^−^.
